# ALDH3A1-dependent Nrf2/HO-1/GPX4 pathway supports AHR as a promising therapeutic target for ferroptosis and promotes imperatorin-mediated lung protection

**DOI:** 10.1038/s41420-025-02860-8

**Published:** 2026-01-09

**Authors:** Xiaominting Song, Wenya Yang, Hang You, Shan Qian, Xiaoxue Hu, Ao Zhang, Jia Li, Yuzhi Li, Huachao Bin, Cheng Peng, Jin Pei, Zhixing Cao

**Affiliations:** 1 State Key Laboratory of Southwestern Chinese Medicine Resources, School of Pharmacy, Chengdu University of Traditional Chinese Medicine, Chengdu, China; 2 Department of Pharmaceutical Engineering, College of Food and Bioengineering, Xihua University, Chengdu, China; 3 Institute of Herbgenomics, Chengdu University of Traditional Chinese Medicine, Chengdu, China

**Keywords:** Respiratory tract diseases, Pharmacology

## Abstract

The aryl hydrocarbon receptor (AHR) is a transcription factor prominently expressed at barrier sites, while aldehyde dehydrogenase 3 family member A1 (ALDH3A1) is a metabolic enzyme implicated in oxidative stress. However, their roles in ferroptosis remain poorly understood. Imperatorin (IMP) is a bioactive compound derived from traditional Chinese medicine. Here, we demonstrate that IMP is a natural agonist of AHR, inhibiting LPS-induced ferroptosis, inflammation, and barrier damage in lung epithelial cells by promoting AHR nuclear translocation and activation. Mechanistically, IMP-activated AHR stimulated the Nrf2/HO-1/GPX4 axis and enhanced ALDH3A1 expression, thereby inhibiting ferroptosis-related Fe^2+^ accumulation, ROS production, and lipid peroxidation. The in vivo results showed that oral IMP activated the AHR/ALDH3A1 and Nrf2/HO-1/GPX4 pathways in lung tissue, thus improving lung dysfunction and inflammation in acute lung injury (ALI) mice induced by LPS. Notably, ALDH3A1 is a key downstream signaling protein of AHR. An AHR inhibitor reversed the IMP-induced upregulation of ALDH3A1, whereas an ALDH3A1 inhibitor blocked the anti-ferroptotic Nrf2/HO-1/GPX4 pathway and diminished the lung-protective effects of IMP-activated AHR both in vitro and in vivo. These findings indicate that the AHR/ALDH3A1 axis may represent a previously unrecognized therapeutic target for ferroptosis and provide insight into IMP as a therapeutic strategy to prevent and treat ALI.

## Introduction

Acute lung injury (ALI) and acute respiratory distress syndrome (ARDS) represent severe respiratory conditions marked by intense lung inflammation, oxidative stress, epithelial barrier dysfunction, and pulmonary edema. These conditions exhibit a considerable mortality rate, which varies between 30% and 40% [1, 2]. Despite extensive research, protective pulmonary ventilation remains the primary treatment for ALI [3]. The worldwide emergence of coronavirus disease 2019 (COVID-19) has intensified the need for more effective pharmacological treatments for ALI/ARDS [4, 5].

The aryl hydrocarbon receptor (AHR), a ligand-dependent transcription factor expressed in various immune cells, is considered a promising target for regulating both innate and adaptive immunity [6]. Within the traditional AHR signaling pathway, ligand binding induces the movement of the AHR complex to the nucleus, where it partners with the AHR nuclear translocator (ARNT) protein. This interaction produces an AHR-ARNT heterodimer, which attaches to dioxin-responsive elements in genomic areas, thus triggering the expression of several target genes, such as cytochrome P450 (CYP) 1A1, CYP1A2, CYP1B1, and AHR repressor (AHRR) [7]. Notably, the regulation of AHR activity involves three distinct negative-feedback mechanisms: ligand degradation by CYP1A1, the breakdown of AHR-ARNT complexes by AHRR, and AHR degradation through proteasomal pathways [8]. In addition, AHR activation also regulates cytokine production, including interleukin (IL)-10, IL-22, and tumor necrosis factor (TNF)-α, by influencing immune regulatory factors such as nuclear factor-*κ*B (NF-*κ*B), and epidermal growth factor receptor [9]. However, challenges remain with AHR agonists, particularly because increased CYP enzyme activity induced by AHR activation can affect their absorption and entry into the circulatory system [10]. Thus, while AHR represents a promising target for immune modulation, orally bioavailable AHR agonists with improved safety profiles are urgently needed.

Recent research underscores the essential function of AHR in maintaining barrier organ homeostasis (lungs, gut, and skin) and protecting tissue cells from damage by foreign agents [11]. For example, Major et al. demonstrated that AHR is highly expressed (basal levels) in type II alveolar epithelial cells and endothelial cells, with endothelial AHR deficiency causing lung vascular leakage, aggravated lung damage, and increased susceptibility to viral infections [12]. In intestinal pathology, Wiggins et al. identified AHR as a regulator of environmental sensing in gut endothelial cells that is essential for maintaining endothelial quiescence and intestinal homeostasis [13]. Li et al. further showed that baicalein upregulated tight junction proteins (ZO-1 and Occludin) and enhanced IL-22 production by activating AHR, thereby preserving intestinal barrier integrity and immune balance [14]. Dermatologically, activated AHR collaborates with JUN to suppress the production of C-X-C motif ligand 13 by CD^4+^ T cells and promote an IL-22 phenotype, showing therapeutic promise in systemic lupus erythematosus [15]. Furthermore, the AHR agonist tapinarof (Tapi) has demonstrated potential in inhibiting the differentiation of T helper 17 (Th17) cells and Th22 cells and is clinically approved for the topical treatment of plaque psoriasis [16]. Although AHR has been extensively studied in intestinal and skin diseases, its biological function in ALI remains incompletely understood.

Aldehyde dehydrogenase 3 family member A1 (ALDH3A1) is a metabolic enzyme involved in inflammation, oxidative stress, and lipid peroxidation [17, 18]. Inhibiting ALDH3A1 can cause the accumulation of the toxic aldehyde 4-hydroxynonenal within cells. This accumulation results from an imbalance in the glutathione-mediated antioxidant system, which can ultimately affect cell growth [18]. Lipid peroxidation is a crucial metabolic event in ferroptosis, resulting in the generation of lipid peroxidation products such as 4-hydroxynonenal, which contribute to oxidative damage [19]. However, the biological function of ALDH3A1 in ferroptosis requires further investigation. Recent evidence indicates that during ischemia/reperfusion liver injury, the activation of AHR inhibits ferroptosis, a physiological process [20]. However, the protective effects of AHR activation against ferroptosis in ALI, particularly its interaction with ALDH3A1, remain unclear.

In recent years, herbal medicines and functional foods have garnered significant interest for their potential in treating ALI. Imperatorin (IMP) is a high-content active ingredient found in traditional Chinese medicines, including *Angelica dahurica*, *Notopterygium incisum*, *Glehnia littoralis*, and *Clausena lansium*, which are uesd to treat respiratory and pulmonary issues, such as cough, asthma, and influenza [21]. IMP is also a key ingredient in Huoxiang Zhengqi dropping pills, an established remedy for COVID-19 [22]. Notably, IMP has demonstrated therapeutic potential for ALI, primarily due to its anti-inflammatory properties. Previous research showed that IMP alleviated pulmonary inflammation in ALI mice by suppressing cytokine levels through the modulation of the MAPK and NF-*κ*B signaling pathways [23]. However, IMP’s effects on ALI-associated ferroptosis and oxidative damage, as well as its protective role in lung cells, particularly regarding therapeutic targets, remain underexplored.

In this study, we showed that IMP alleviated lipopolysaccharide (LPS)-induced ALI both in vitro and in vivo by exerting anti-ferroptosis, anti-inflammatory, and antioxidant properties. Gene set enrichment analysis of differentially expressed genes (DEGs) indicated that IMP’s lung-protective effects may be achieved by modulating the AHR and ALDH3A1 signaling pathways. Based on these observations, we hypothesize that AHR activation by IMP offers therapeutic benefits for ALI and that ALDH3A1 is a crucial protein mediating AHR ferroptosis inhibition and lung protection. Thus, we experimentally investigated the mechanistic interplay between IMP, AHR, ALDH3A1, and ferroptosis in ALI to validate this hypothesis.

## Results

### IMP protects LPS-induced lung epithelial cells injury

**Fig. 1:**
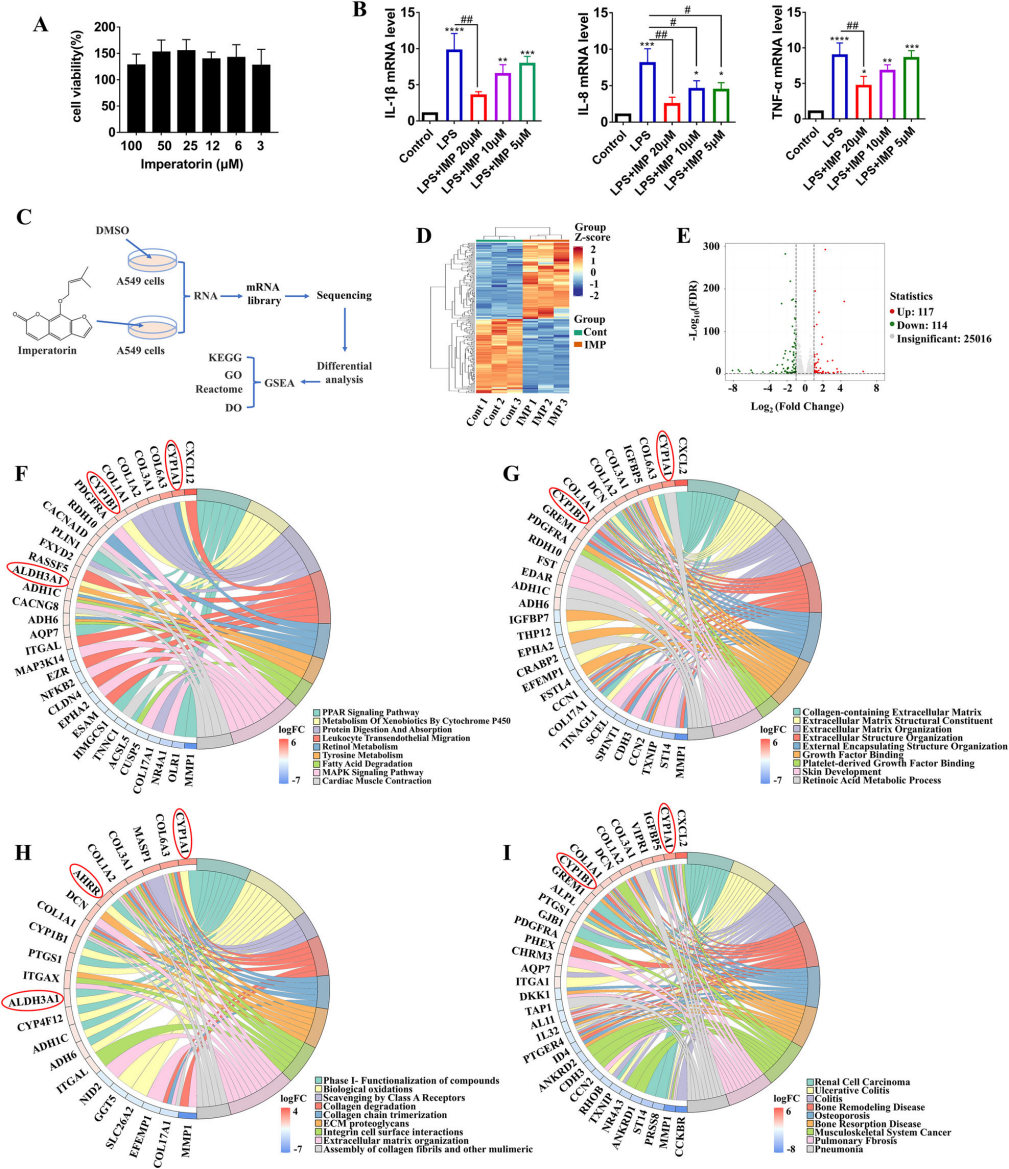
Protective effect of IMP on LPS-induced lung epithelial cells is related to AHR and ALDH3A1 signaling. **A** Viability of A549 cells treated with IMP for 24 h. **B** mRNA expression levels of the inflammatory factors IL-1β, IL-8, and TNF-α in LPS (10 μg/mL)-induced A549 cells treated with IMP for 24 h. **C** Schematic diagram illustrating the RNA-seq analysis in A549 cells treated with DMSO (1%) and IMP (20 μM) for 24 h. **D** Heatmap displaying the overall distribution of DEGs, with red indicating the upregulated genes and blue indicating the downregulated genes. **E** Volcano plot showing the DEGs in the IMP group versus the control group. **F** Chord plot of KEGG enrichment analysis. **G** Chord plots of GO enrichment analysis. **H** Chord plot of Reactome enrichment analysis. **I** Chord plot of DO enrichment analysis. Data are expressed as mean ± SD (*n* = 3). *p < 0.05, **p < 0.01, ***p < 0.001, ****p < 0.0001, versus control group; ^#^*p* < 0.05, ^##^*p* < 0.01, versus LPS group.

Lung epithelial cells are crucial for coordinating host defenses and maintaining lung homeostasis. They actively participate in various pathological conditions, including ARDS, viral infections, and other respiratory diseases [24]. This study first established a 10 μg/mL LPS-induced A549 lung epithelial cell model to preliminarily investigate the pulmonary protective effects of IMP according to a previous publication [25]. The results indicated that IMP was not cytotoxic to A549 cells in the concentration range of 0.4–100 μM (Fig. 1A). Further analysis of intermediate concentrations of IMP (20, 10, and 5 μM) revealed that it significantly inhibited the release of inflammatory cytokines (IL-1β, IL-8, and TNF-α), thereby attenuating LPS-induced inflammatory damage in lung epithelial cells (Fig. 1B).

### RNA-seq reveals IMP-induced overexpression of AHR and ALDH3A1 signaling in lung epithelial cells

To clarify the potential mechanisms underlying IMP’s protective effects on LPS-induced lung epithelial cells, transcriptome sequencing was performed on both control A549 cells and 20 μM IMP-treated cells (Fig. 1C). DEG analysis, illustrated by heatmaps and volcano plots, revealed 117 upregulated genes in the IMP group compared to the control group, while 114 genes were downregulated (Fig. 1D, E). Enrichment analyses using the Kyoto Encyclopedia of Genes and Genomes (KEGG), Gene Ontology (GO), Reactome, and Disease Ontology (DO) databases identified nine pathways with the most significant q-values, which were subsequently visualized using chord plots. IMP-mediated DEGs showed significant enrichment in pathways related to “cytochrome P450,” “tyrosine metabolism,” “retinoic acid metabolic process,” “biological oxidations,” and “pneumonia.” Notably, among the DEGs associated with these pathways, the *CYP1A1*, *CYP1B1*, *AHRR*, and *ALDH3A1* genes were markedly upregulated by IMP (Fig. 1F–I). CYP1A1, CYP1B1, and AHRR serve as defining downstream targets within the AHR pathway, where the expression of CYP1A1 highly relies on AHR activity and is significantly enhanced upon AHR activation [26]. Thus, the aforementioned findings suggest that the protective effect of IMP on A549 cells may be attributed to its modulation of AHR and ALDH3A1 signaling pathways.

### IMP triggers AHR nuclear translocation in lung epithelial cells

**Fig. 2:**
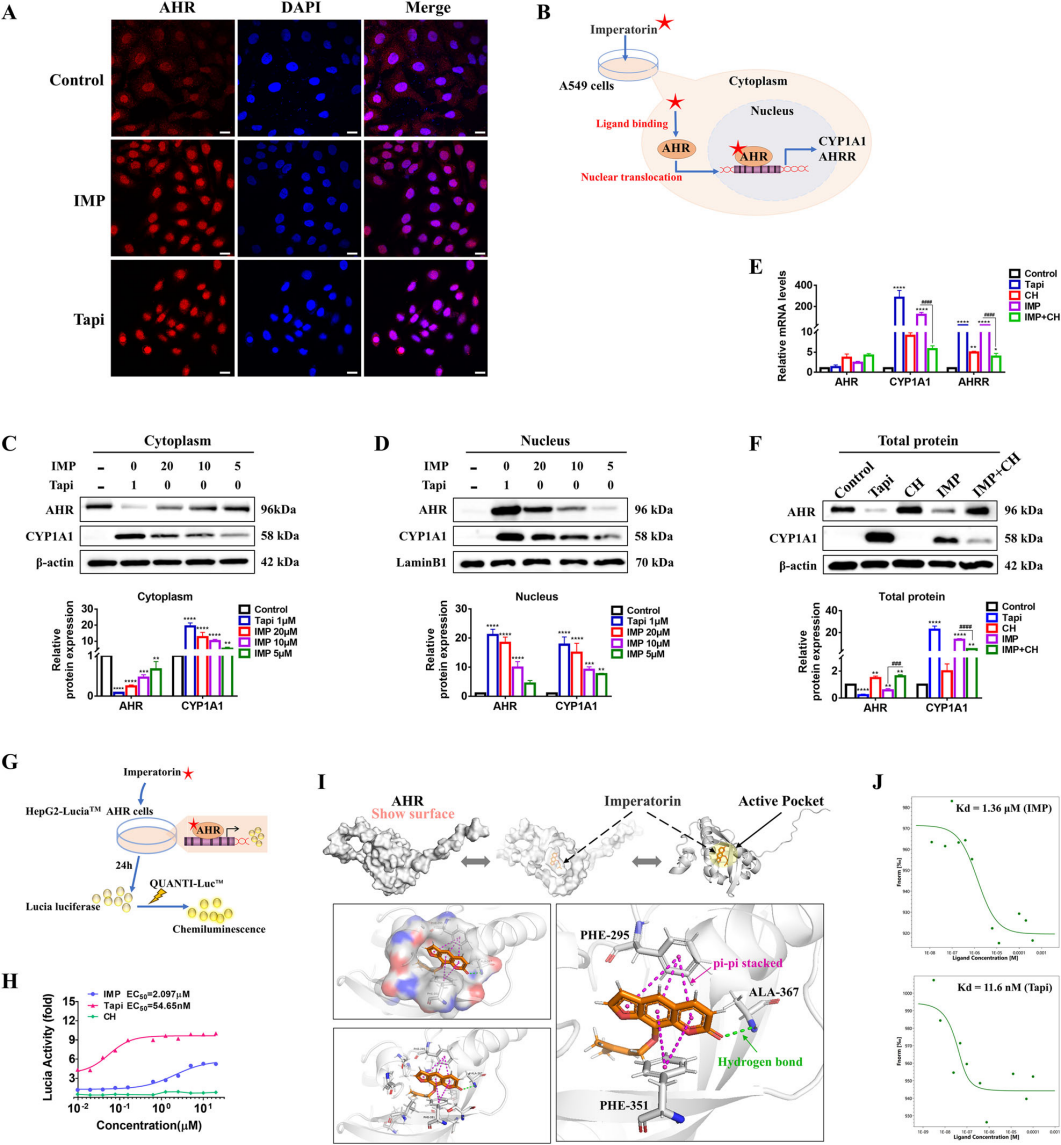
IMP triggers AHR nuclear translocation-transcriptional activation in A549 cells. **A** Immunofluorescence showing the intracellular localization of AHR in A549 cells treated with IMP (20 μM) or Tapi (1 μM) for 24 h (scale bar = 20 μm). **B** Schematic diagram of AHR nuclear translocation triggered by IMP in A549 cells. **C**, **D** Western blotting analysis of AHR and CYP1A1 in the cytoplasm and nucleus of A549 cells. **E** mRNA expression levels of AHR, CYP1A1, and AHRR in A549 cells. **F** Western blotting analysis of AHR and CYP1A1 in total protein of A549 cells. Concentrations in (**E**, **F**): IMP, 20 μM; Tapi, 1 μM; CH, 10 μM. **G** Schematic diagram of compound activity using the AHR-driven luciferase reporter assay in HepG2-Lucia™ AHR reporter cells. **H** Effect of IMP, Tapi, and CH on AHR. **I** Molecular docking results of IMP with AHR. **J** The MST assay measured the Kd values for the interaction between AHR and IMP or Tapi. Data are expressed as mean ± SD (*n* = 3). *p < 0.05, **p < 0.01^,^ ***p < 0.001, ****p < 0.0001, versus control group; ^###^*p* < 0.001, ^####^*p* < 0.0001, versus IMP group.

To confirm whether IMP could trigger AHR activation in lung epithelial cells, A549 cells were exposed to IMP for 24 h. Consistent with AHR activation, immunofluorescence analysis revealed that IMP promoted AHR translocation from the cytoplasm to the nucleus, an effect comparable to that in the positive control Tapi (Fig. 2A). At the protein level, IMP downregulated AHR expression in the cytoplasm while upregulating it in the nucleus in a concentration-dependent manner. It also significantly enhanced CYP1A1 expression in both cellular compartments. This pattern was consistent with that observed for Tapi (Fig. 2B–D).

Subsequently, in order to evaluate the targeting of IMP to AHR, cells were treated with the AHR inhibitor CH223191 (CH). Similar to Tapi, IMP significantly upregulated CYP1A1 and AHRR at transcriptional (mRNA) and translational (protein) levels. However, CH reversed this trend, indicating that IMP’s regulation of CYP1A1 and AHRR is mediated by AHR (Fig. 2E, F). Notably, treatment with IMP or Tapi decreased intracellular AHR protein expression in A549 cells, which corresponds to the canonical response upon AHR activation. This phenomenon is mechanistically regulated by negative feedback control mediated by the downstream targets CYP1A1 and AHRR [7, 8]. Collectively, these findings suggest that IMP triggers AHR nuclear translocation-transcriptional activation in lung epithelial cells, significantly upregulating its downstream target genes CYP1A1 and AHRR.

### Agonistic activity and binding affinity of IMP to AHR

To further assess the agonistic activity of IMP on AHR, luciferase activity was measured in HepG2 Lucia™ AHR reporter cells. These cells were genetically engineered to express an AHR-mediated Lucia luciferase reporter gene plasmid (Fig. 2G) [27]. IMP exhibited moderate AHR agonistic activity, with an EC_50_ value (concentration for 50% of maximal effect) of 2.097 μM. Notably, 5 μM IMP enhanced AHR activity approximately five-fold and sustained AHR activation, even between 0.1 and 0.01 μM (Fig. 2H). In comparison, Tapi demonstrated significantly stronger agonistic activity, achieving a ten-fold enhancement. However, persistent AHR activation can adversely affect immune defense mechanisms, induce cell dedifferentiation, promote tumor immune evasion, and contribute to therapeutic resistance [28]. Next, molecular docking analysis revealed the binding affinity between IMP and AHR, with a calculated binding energy of -9.1 kcal/mol. Regarding the binding mode, IMP interacted with the AHR protein by establishing a hydrogen bond with the ALA-367 residue and engaging in π-π stacking interactions with residues PHE-295 and PHE-351 (Fig. 2I). To further validate the docking results, the binding constant (Kd value) between AHR and IMP was determined using microscale thermophoresis (MST). IMP exhibited a robust binding affinity for AHR with a Kd value of 1.36 μM. The positive control, Tapi, had a Kd value of 11.6 nM. (Fig. 2J). Taken together, the luciferase reporter assay, molecular docking, and MST data collectively indicate that IMP interacts favorably with AHR.

### IMP inhibits ferroptosis in LPS-induced lung epithelial cells

**Fig. 3:**
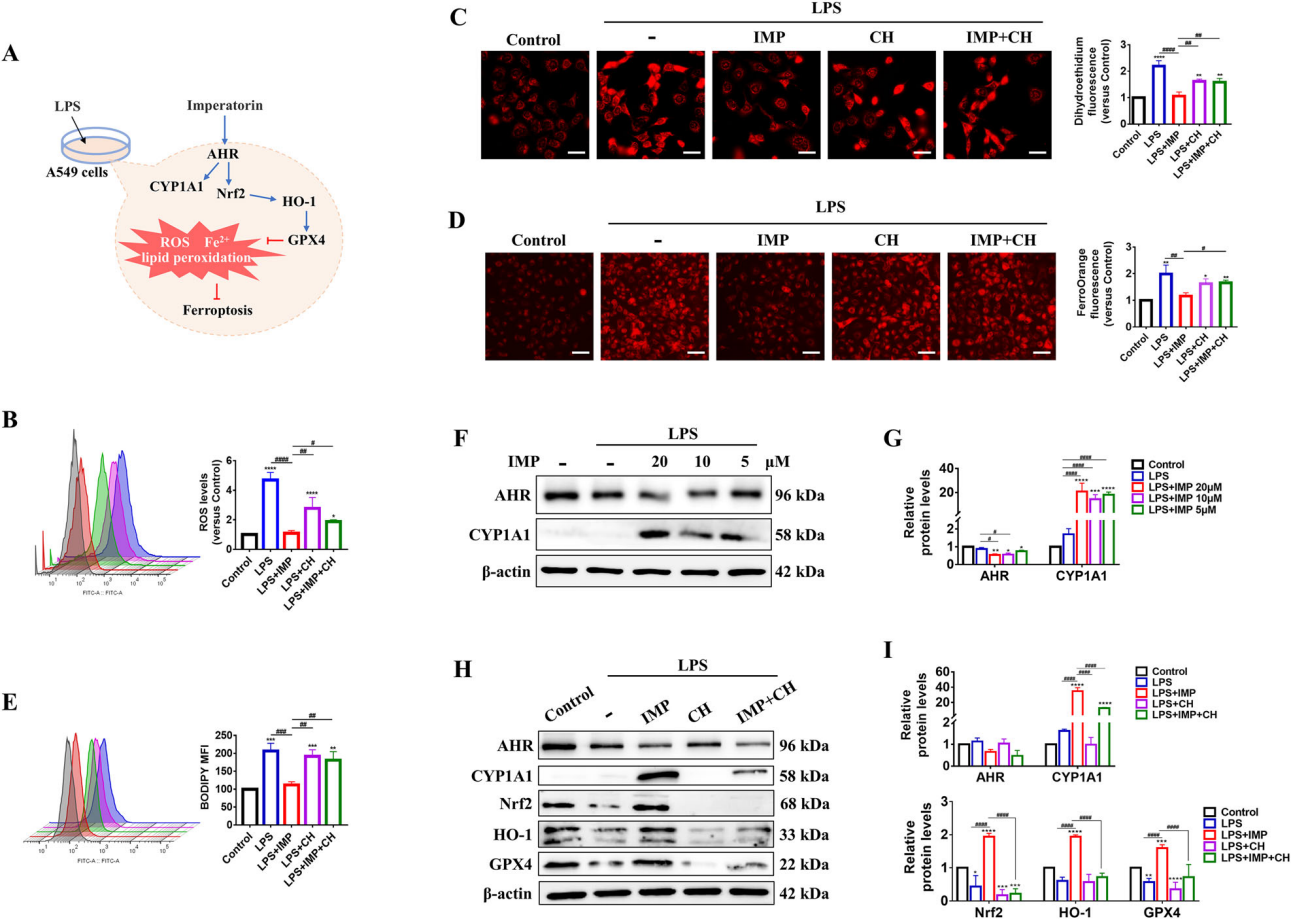
IMP inhibits ferroptosis in LPS-induced A549 cells by regulating the Nrf2/HO-1/GPX4 axis through AHR activation. **A** Schematic diagram showing ferroptosis inhibition in LPS (10 μg/mL)-induced A549 cells treated with IMP (20 μM) for 24 h. **B** Representative flow cytometry and statistical analysis of DCFH-DA fluorescence indicating the ROS levels. **C** Representative images of DHE fluorescence and the statistical analysis of intracellular superoxide anion levels (scale bar = 25 μm). **D** Representative images of FerroOrange fluorescence and the statistical analysis of Fe^2+^ levels (scale bar = 50 μm). **E** Representative flow cytometry and statistical analysis of BODIPY lipid peroxidation assay. **F**, **G** Western blotting analysis of AHR and CYP1A1 proteins in LPS-induced A549 cells. **H**, **I** Western blotting analysis of AHR, CYP1A1, Nrf2, HO-1, and GPX4 proteins in LPS-induced A549 cells. The AHR inhibitor CH (10 μM) was used to study the AHR dependence of IMP's anti-ferroptotic effect. Data are expressed as mean ± SD (*n* = 3). *p < 0.05, ^**^p < 0.01, ***p < 0.001, ****p < 0.0001, versus control group; ^#^*p* < 0.05, ^##^*p* < 0.01, ^###^*p* < 0.001, ^####^*p* ^<^ 0.0001, versus LPS + IMP or LPS group.

Ferroptosis features Fe²⁺ accumulation and lipid peroxidation, where reactive oxygen species (ROS) act as key drivers of oxidative stress and lipid damage [29]. IMP’s impact on LPS-induced ferroptosis in lung epithelial cells was assessed by measuring ROS, Fe²⁺, and lipid peroxidation levels (Fig. 3A). DCFH-DA staining showed that IMP treatment significantly attenuated LPS-induced increases in ROS levels (Fig. 3B). Dihydroethidium (DHE) fluorescent probe staining further confirmed that IMP ameliorated LPS-induced elevation of superoxide anions in A549 cells, as indicated by reduced red fluorescence intensity (Fig. 3C). Notably, the AHR inhibitor CH markedly abolished the suppressive effect of IMP on LPS-induced ROS and superoxide anion levels, suggesting that AHR is critical for the IMP-mediated oxidative defense (Fig. 3B, C). Additionally, FerroOrange fluorescent probe assays revealed elevated Fe²⁺ levels in LPS-stimulated A549 cells, which were reduced by IMP treatment. This effect was reversed by CH treatment (Fig. 3D). Next, BODIPY probe staining further confirmed that LPS triggered lipid peroxidation in A549 cells, while IMP significantly decreased the mean fluorescence intensity (MFI) emitted by the probe at 510 nm, indicating an inhibition of lipid peroxidation and a decrease in lipid ROS accumulation. Similarly, CH treatment reversed the effect of IMP (Fig. 3E). Collectively, these results indicate that IMP inhibits LPS-induced ferroptosis in lung epithelial cells by activating AHR.

### IMP regulates the Nrf2/HO-1/GPX4 axis by activating AHR in response to ferroptosis

This study further investigated whether IMP inhibited ferroptosis in LPS-induced lung epithelial cells by activating AHR. We found that AHR was in an inactivated state in LPS-stimulated A549 cells. IMP treatment dose-dependently activated AHR, resulting in the significant upregulation of the AHR-responsive gene CYP1A1. Decreases in AHR protein levels could be explained by a negative feedback mechanism after AHR activation (Fig. 3F, G). In addition, the nuclear factor erythroid 2-related factor 2/heme oxygenase 1/glutathione peroxidase 4 (Nrf2/HO-1/GPX4) axis has been identified as a critical signaling pathway suppressing ferroptosis by inhibiting ROS and Fe²⁺ generation [30]. Western blotting analysis showed that LPS significantly suppressed Nrf2, HO-1, and GPX4 protein expression in A549 cells, whereas IMP treatment effectively reversed this downregulation. To further validate whether AHR signaling mediates IMP’s regulatory effects on the Nrf2/HO-1/GPX4 axis, A549 cells were co-treated with the AHR inhibitor CH. CH markedly blocked the IMP-induced upregulation of these proteins in LPS-exposed cells (Fig. 3H, I). These findings demonstrate that IMP activates the Nrf2/HO-1/GPX4 pathway through AHR-dependent mechanisms, thereby inhibiting LPS-induced ferroptosis in lung epithelial cells.

### ALDH3A1 is essential for AHR to activate the Nrf2/HO-1/GPX4 axis

**Fig. 4:**
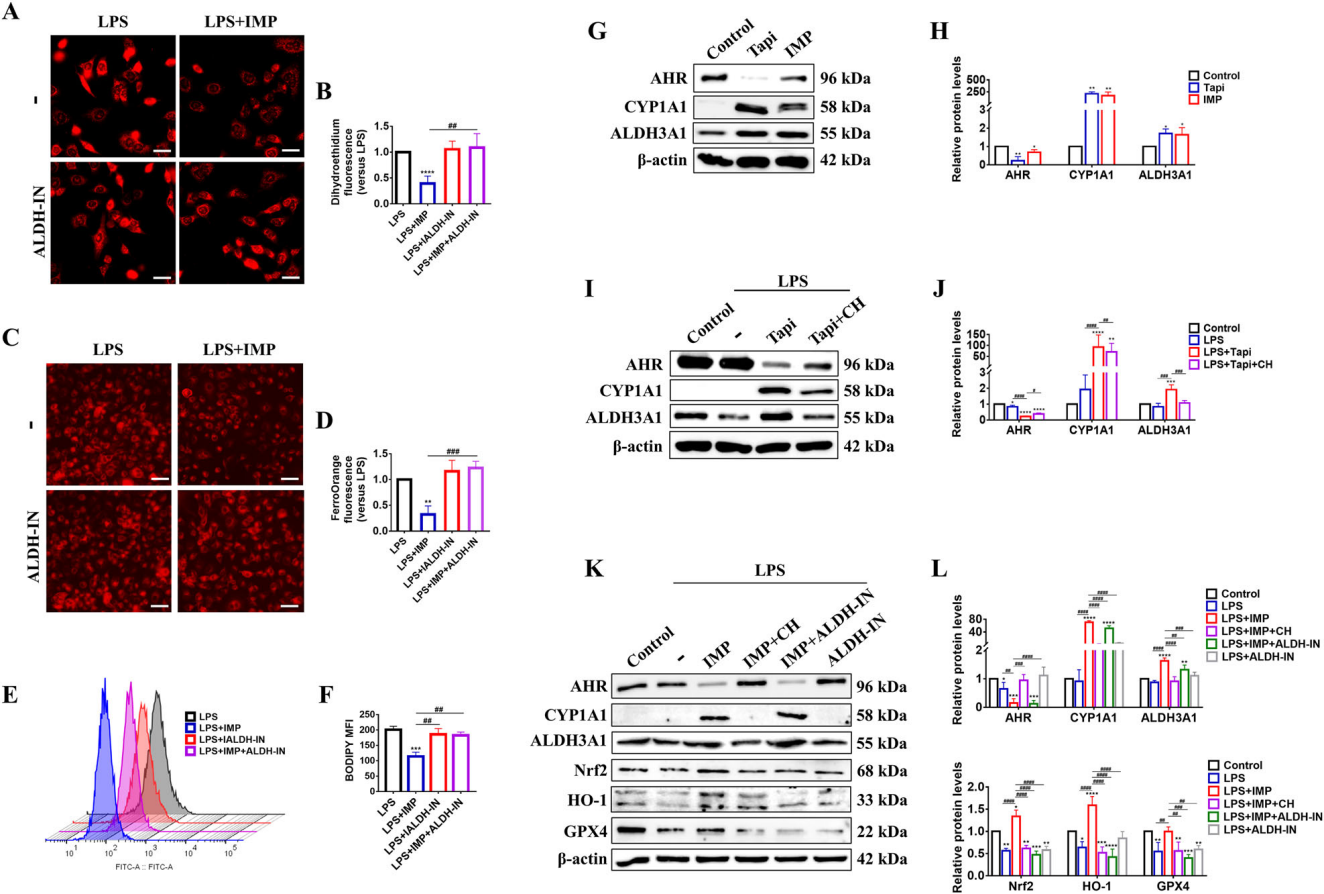
ALDH3A1 is a key protein in the AHR regulation of the Nrf2/HO-1/GPX4 axis. **A**, **B** Representative images of DHE fluorescence and the statistical analysis of intracellular superoxide anion levels (scale bar = 25 μm). **C**, **D** Representative images of FerroOrange fluorescence and the statistical analysis of Fe^2+^ levels (scale bar = 50 μm). **E**, **F** Representative flow cytometry and statistical analysis of BODIPY lipid peroxidation assay. **G**, **H** Western blotting analysis of AHR, CYP1A1, and ALDH3A1 proteins in A549 cells. **I**, **J** Western blotting analysis of AHR, CYP1A1, and ALDH3A1 proteins in LPS-induced A549 cells. **K**, **L** Western blotting analysis of AHR, CYP1A1, ALDH3A1, Nrf2, HO-1, and GPX4 proteins in LPS-induced A549 cells. Concentrations: LPS, 10 μg/mL; IMP, 20 μM; Tapi, 1 μM; CH, 10 μM; ALDH-IN, 10 μM. Data are expressed as mean ± SD (*n* = 3). *p < 0.05, **p < 0.01, ***p < 0.001, ****p < 0.0001, versus control group; ^##^*p* < 0.01, ^###^*p* < 0.001, ^####^*p* < 0.0001, versus LPS + IMP or LPS + Tapi group.

Building on RNA-seq analysis, IMP also significantly upregulated ALDH3A1 expression in A549 lung epithelial cells (Fig. 1F, H). Therefore, this study further explored the biological function of ALDH3A1. Treating A549 cells with ALDH3A1-IN-1 (ALDH-IN), a specific pharmacological inhibitor of ALDH3A1, significantly counteracted the inhibitory effects of IMP on ferroptosis markers, including ROS production, Fe²⁺ accumulation, and lipid peroxidation, in LPS-challenged A549 cells (Fig. 4A–F). The results indicate that ALDH3A1, like AHR, serves as a key mediator for IMP in mitigating ferroptosis in lung epithelial cells. Next, we investigated the regulatory cascade between ALDH3A1 and AHR. The results showed that both the AHR-specific agonist Tapi and IMP not only induced AHR activation in A549 cells, but also upregulated the expression of ALDH3A1 (Fig. 4G, H). Furthermore, in LPS-induced A549 cells, Tapi-upregulated ALDH3A1 levels were significantly reversed by the AHR-specific inhibitor CH, indicating that ALDH3A1 protein expression is regulated by AHR (Fig. 4I, J).

Next, we examined whether ALDH3A1 participates in the AHR-mediated regulation of the Nrf2/HO-1/GPX4 axis. The results showed that in LPS-induced A549 cells, CH blocked IMP-induced AHR activation and ALDH3A1 upregulation, whereas ALDH-IN did not alter AHR activity. Notably, ALDH-IN significantly abolished IMP-induced activation of the AHR-dependent Nrf2/HO-1/GPX4 pathway (Fig. 4K, L). Taken together, these results indicate that ALDH3A1 serves as a key mediator linking AHR to the Nrf2/HO-1/GPX4 axis, thereby contributing to IMP’s pulmonary protective effects. Mechanistically, IMP binds to and activates AHR in lung epithelial cells, which subsequently induces ALDH3A1 activation. This cascade ultimately upregulates the Nrf2/HO-1/GPX4 axis to mitigate LPS-induced ferroptosis.

### IMP inhibits LPS-induced inflammation and epithelial barrier damage in lung epithelial cells by activating AHR

**Fig. 5:**
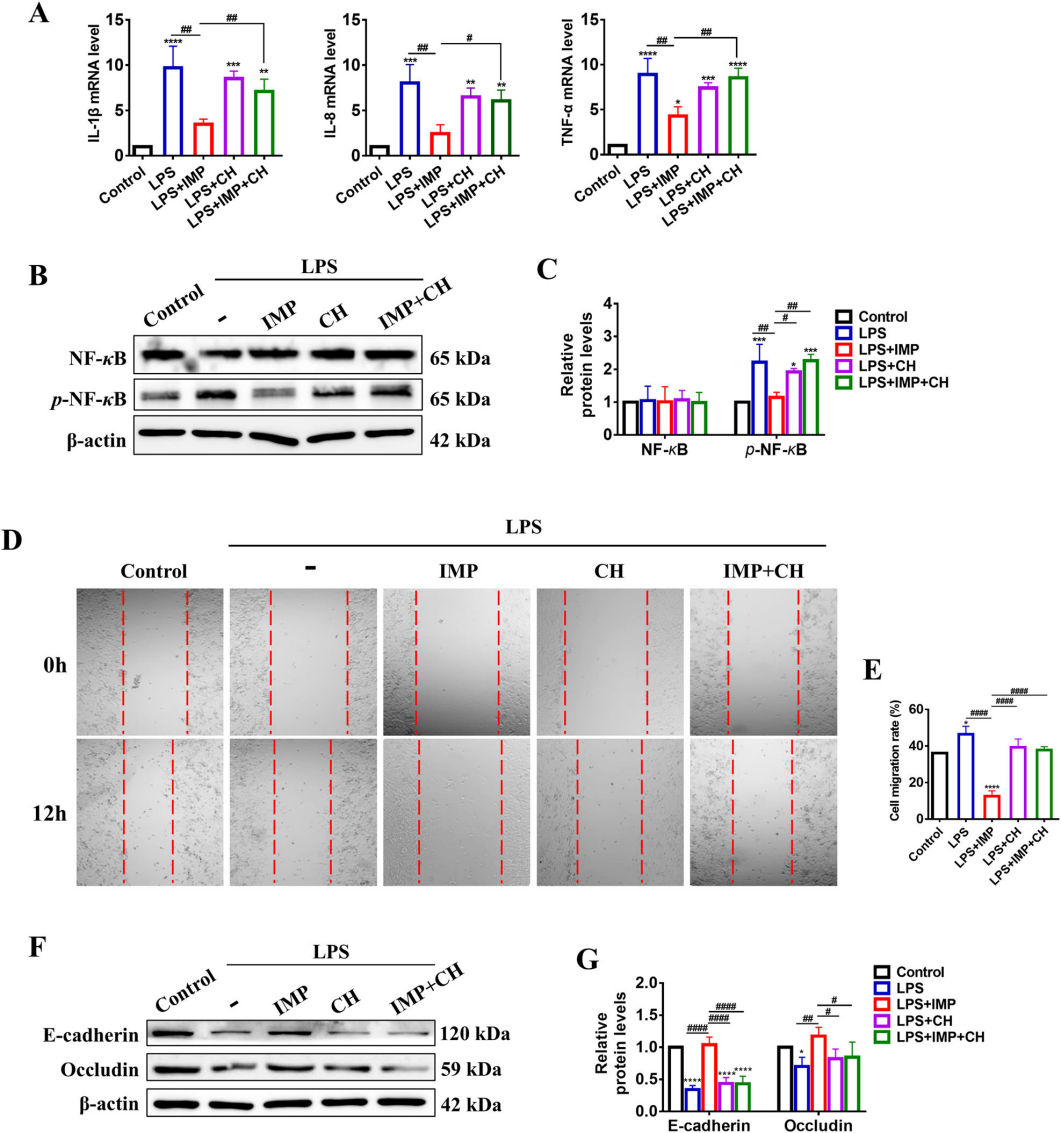
IMP inhibits inflammation and epithelial barrier damage in LPS-induced A549 cells by activating AHR. **A** mRNA expression levels of IL-1β, IL-8, and TNF-α in A549 cells. **B**, **C** Western blotting analysis of NF-*κ*B and *p*-NF-*κ*B proteins in LPS-induced A549 cells. **D**, **E** Representative images and quantitative analysis of cell migration (200× magnification). **F**, **G** Western blotting analysis of E-cadherin and Occludin proteins in LPS-induced A549 cells. Concentrations: LPS, 10 μg/mL; IMP, 20 μM; Tapi, 1 μM; CH, 10 μM. Data are expressed as mean ± SD (*n* = 3). *p < 0.05, **p < 0.01, ***p < 0.001, ****p < 0.0001, versus control group; ^#^*p* < 0.05, ^##^*p* < 0.01, ^####^*p* < 0.0001, versus LPS + IMP group.

The inflammatory response is the primary pathological hallmark of ALI and is characterized by the release of multiple inflammatory mediators. In LPS-stimulated A549 cells, IMP significantly downregulated mRNA levels of IL-1β, IL-8, and TNF-α. This effect was prevented by the AHR inhibitor CH, demonstrating that IMP’s anti-inflammatory activity is AHR-dependent (Fig. 5A). As NF-*κ*B activation represents a pivotal pathway for inflammatory factor release [31], we further examined its phosphorylation status. Western blotting analysis revealed that IMP suppressed LPS-induced NF-*κ*B phosphorylation, an effect abolished by CH co-treatment (Fig. 5B, C). These findings collectively demonstrate that AHR regulates NF-*κ*B transcriptional activation, and that IMP alleviates inflammation in LPS-stimulated lung epithelial cells through the AHR-dependent suppression of NF-*κ*B activation.

Excessive inflammation and oxidative stress are widely recognized as critical contributors to epithelial barrier injury. To assess IMP’s protection against LPS-induced epithelial barrier damage, wound healing assays were performed. LPS significantly accelerated A549 cell migration versus controls, whereas IMP treatment suppressed this migration. Importantly, co-treatment with the AHR inhibitor CH reversed IMP’s inhibitory effect, confirming that AHR mediates IMP’s protection of barrier integrity (Fig. 5D, E). Subsequently, we further analyzed the expression of E-cadherin and Occludin, two key proteins governing tight junction formation and epithelial barrier integrity [32, 33]. IMP treatment significantly counteracted the LPS-induced downregulation of both E-cadherin and Occludin protein levels. However, this restorative effect was abolished when LPS-induced A549 cells were co-treated with CH and IMP (Fig. 5F, G). These findings collectively demonstrate that IMP enhances E-cadherin and Occludin protein expression in LPS-induced lung epithelial cells by activating AHR, thereby preserving epithelial barrier function.

### IMP activates lung AHR and improves lung shadow and pulmonary dysfunction in ALI mice

**Fig. 6:**
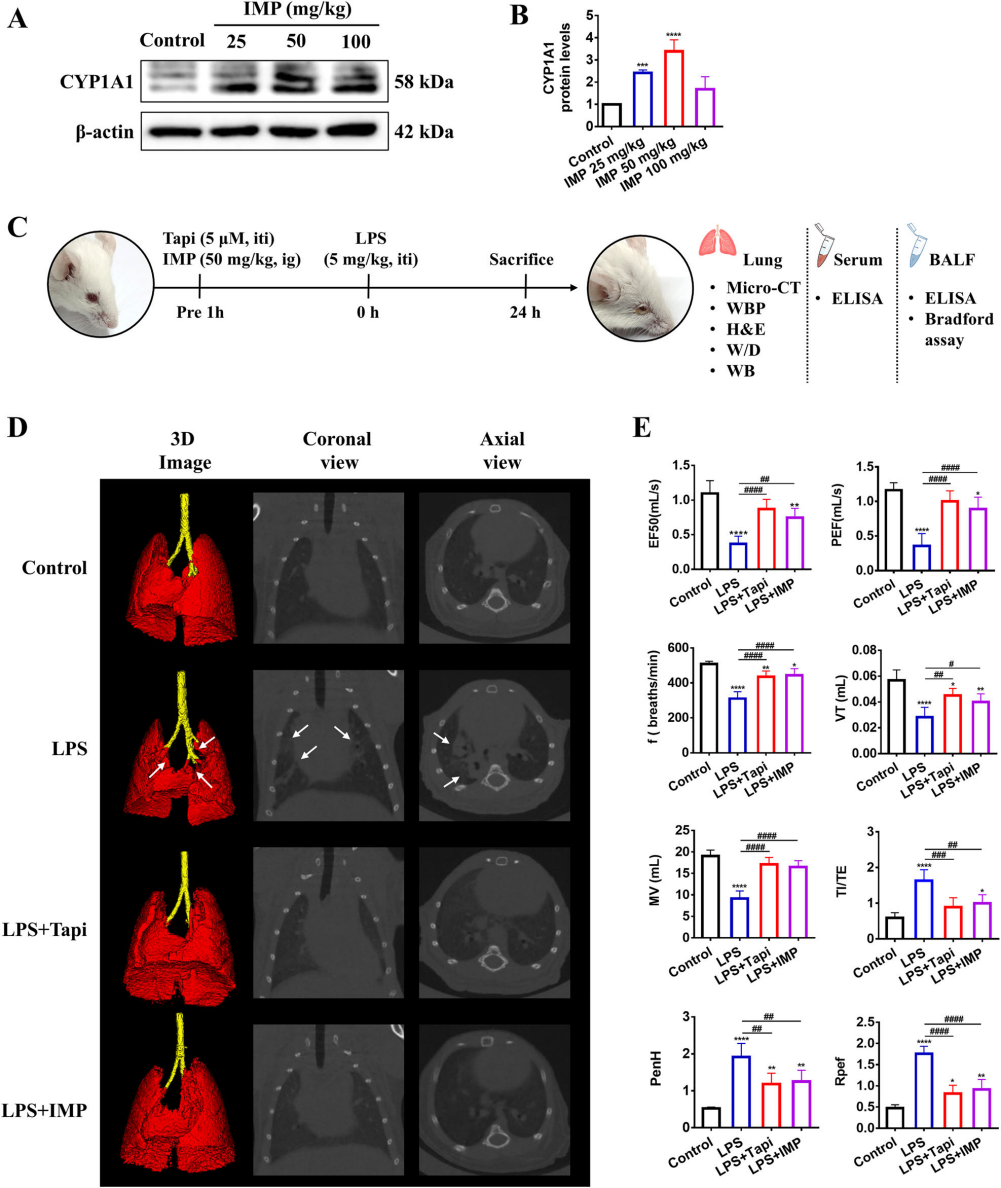
IMP activates AHR in mouse lung tissue and improves lung shadows and pulmonary dysfunction in LPS-induced ALI mice. **A**, **B** Western blotting analysis of CYP1A1 protein in the lung tissues of mice 1 h after the oral administration of IMP (*n* = 3). **C** Schematic diagram of IMP and Tapi treatment in LPS-induced ALI mice. **D** Representative images of 3D lung reconstruction in mice, including both coronal and axial micro-CT views (*n* = 6). **E** Lung function indicators in mice: EF50, PEF, f, VT, MV, TI/TE, PenH, and Rpef (*n* = 6). Abbreviations: ig intragastric gavage, iti intratracheal instillation. Data are expressed as mean ± SD. *p < 0.05, **p < 0.01, ***p < 0.001, ****p < 0.0001, versus control group; ^#^*p* < 0.05, ^##^*p* < 0.01, ^###^*p* < 0.001, ^####^*p* < 0.0001, versus LPS group.

This study already found that IMP activated AHR in lung epithelial cells in vitro. Thus, the effect of IMP on endogenous lung AHR was further evaluated in vivo by orally administering varying doses of IMP (25, 50, and 100 mg/kg) to mice. CYP1A1 protein levels in lung tissue, a canonical AHR activation marker, were detected 1 h post-treatment. All IMP doses upregulated CYP1A1 expression, with the most pronounced effect observed at 50 mg/kg (Fig. 6A, B). Based on these findings, 50 mg/kg IMP was selected for subsequent in vivo efficacy studies in LPS-induced ALI mice (Fig. 6C).

The main clinical features of ALI are bilateral diffuse infiltrative shadows and respiratory distress. Therefore, we conducted in vivo micro-CT imaging and whole-body plethysmography (WBP) to evaluate the therapeutic effects of IMP on lung imaging and pulmonary dysfunction in ALI mice. Micro-CT revealed significant defects in the 3D imaging of lung tissue 24 h after LPS stimulation by tracheal nebulization, particularly at the bronchial bifurcation, as indicated by the white arrow. Images from high-resolution X-ray tomography further showed pronounced infiltration shadows in the lung lobes of LPS-infected mice. However, the oral IMP administration and Tapi atomization effectively reduced pulmonary infiltrative shadows and restored lung density to a level comparable to the control group (Fig. 6D). WBP, a non-invasive technique for evaluating lung function, tracked key pulmonary metrics and demonstrated deteriorating pulmonary function in LPS-stimulated mice. Symptoms such as dyspnea, fatigue, weakness, and shortness of breath were evident in the mice. These changes were marked by decreases in expiratory flow at 50% tidal volume (EF_50_), peak expiratory flow (PEF), respiratory frequency (f), tidal volume (VT), and minute ventilation (MV), along with increases in inspiratory-to-expiratory ratio (TI/TE), enhanced pause (PenH), and ratio of peak expiratory flow (Rpef). IMP treatment reversed these adverse trends, improving pulmonary function in ALI mice. Similarly, the AHR agonist Tapi also exhibited therapeutic benefits for pulmonary dysfunction (Fig. 6E).

### IMP attenuates lung structural damage and inflammation in LPS-induced ALI mice by activating AHR/ALDH3A1

**Fig. 7:**
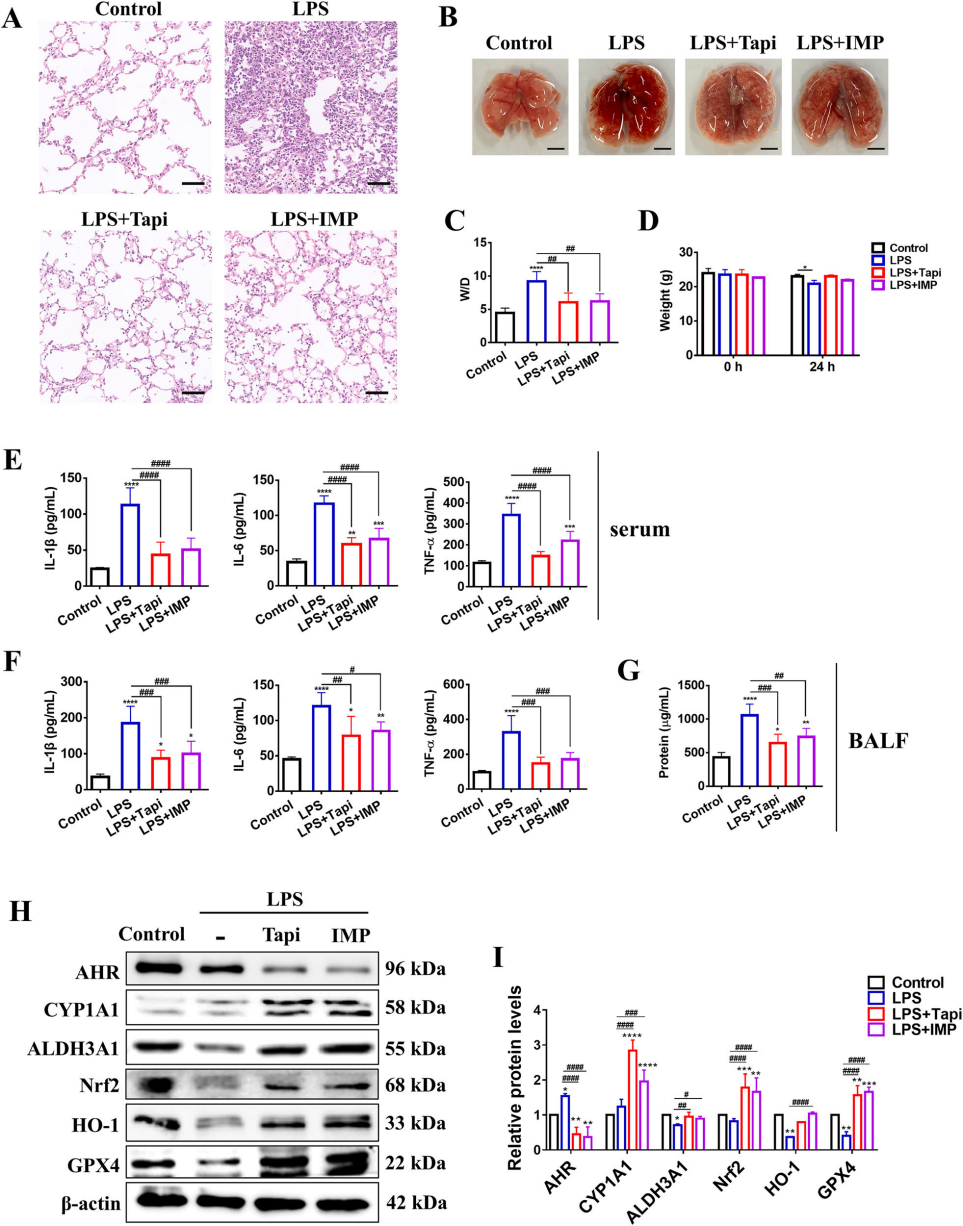
IMP improves lung structural damage and inflammation in LPS-induced ALI mice by regulating the AHR/ALDH3A1 pathway. **A** Representative H&E‑stained lung tissue sections (scale bar = 50 μm). **B** Images of fresh mouse lung tissue (scale bar = 3 mm). **C** Lung W/D ratio. **D** Weight. **E** Serum concentrations of IL-1β, IL-6, and TNF-α. **F** BALF concentrations of IL-1β, IL-6, and TNF-α. **G** Total protein concentrations in BALF. *n* = 6 in all assays depicted in (**A–G**). **H**, **I** Western blotting analysis of AHR/CYP1A1 and Nrf2/HO-1/GPX4 pathways in lung tissue (*n* = 3). Data are expressed as mean ± SD. *p < 0.05, **p < 0.01, ***p < 0.001, ****p < 0.0001, versus control group; ^#^*p* < 0.05, ^##^*p* < 0.01, ^###^*p* < 0.001, ^####^*p* < 0.0001, versus LPS group.

This study then systematically evaluated the therapeutic effect of IMP on LPS-induced ALI in mice, including structural damage manifested by alveolar barrier disruption and pulmonary edema, and dysregulated inflammatory cascades characterized by excessive cytokine production. Hematoxylin and eosin (H&E) staining revealed significant alveolar collapse, thickened alveolar walls, and inflammatory cell infiltration in LPS-infected mice, successfully recapitulating ALI pathological changes. The oral administration of 50 mg/kg IMP ameliorated the above lung pathological changes, with efficacy comparable to that of the AHR agonist Tapi (Fig. 7A). Furthermore, both IMP and Tapi treatments significantly alleviated pulmonary hyperemia and edema, reduced lung wet-to-dry (W/D) ratios, and mitigated body weight loss in ALI mice (Fig. 7B–D). Next, ELISA was used to quantify cytokine levels in the serum and bronchoalveolar lavage fluid (BALF) of ALI mice. As shown in Fig. 7E, F, IMP effectively suppressed LPS-induced elevations in IL-1β, IL-6, and TNF-α in both serum and BALF. Concurrently, BALF supernatant total protein, a key marker for evaluating lung inflammation in ALI [34], was markedly increased by LPS compared to the control group. This increase was prevented by IMP or Tapi treatment (Fig. 7G). Collectively, these results indicate that IMP effectively preventes LPS-induced ALI in vivo by attenuating pulmonary pathology, edema, and inflammation.

To further validate whether IMP ameliorated ALI via the AHR-ALDH3A1-Nrf2/HO-1/GPX4 axis in vivo, we analyzed AHR, ALDH3A1, Nrf2, HO-1, and GPX4 expression in lung tissues by Western blotting. Similar to the in vitro experiment, IMP activated pulmonary AHR in ALI mice, as indicated by upregulated CYP1A1. The observed AHR downregulation may be attributed to the initiation of a negative feedback mechanism that avoids the toxic effects of sustained AHR activation. Furthermore, IMP treatment reversed LPS-induced decreases in ALDH3A1, Nrf2, HO-1, and GPX4 protein levels (Fig. 7H, I). These findings suggest that IMP could improve ALI in vivo by activating AHR and its downstream ALDH3A1-Nrf2/HO-1/GPX4 signaling pathway in lung tissue.

### AHR or ALDH3A1 inhibitors abolish the ALI-protective effect of IMP in vivo

**Fig. 8:**
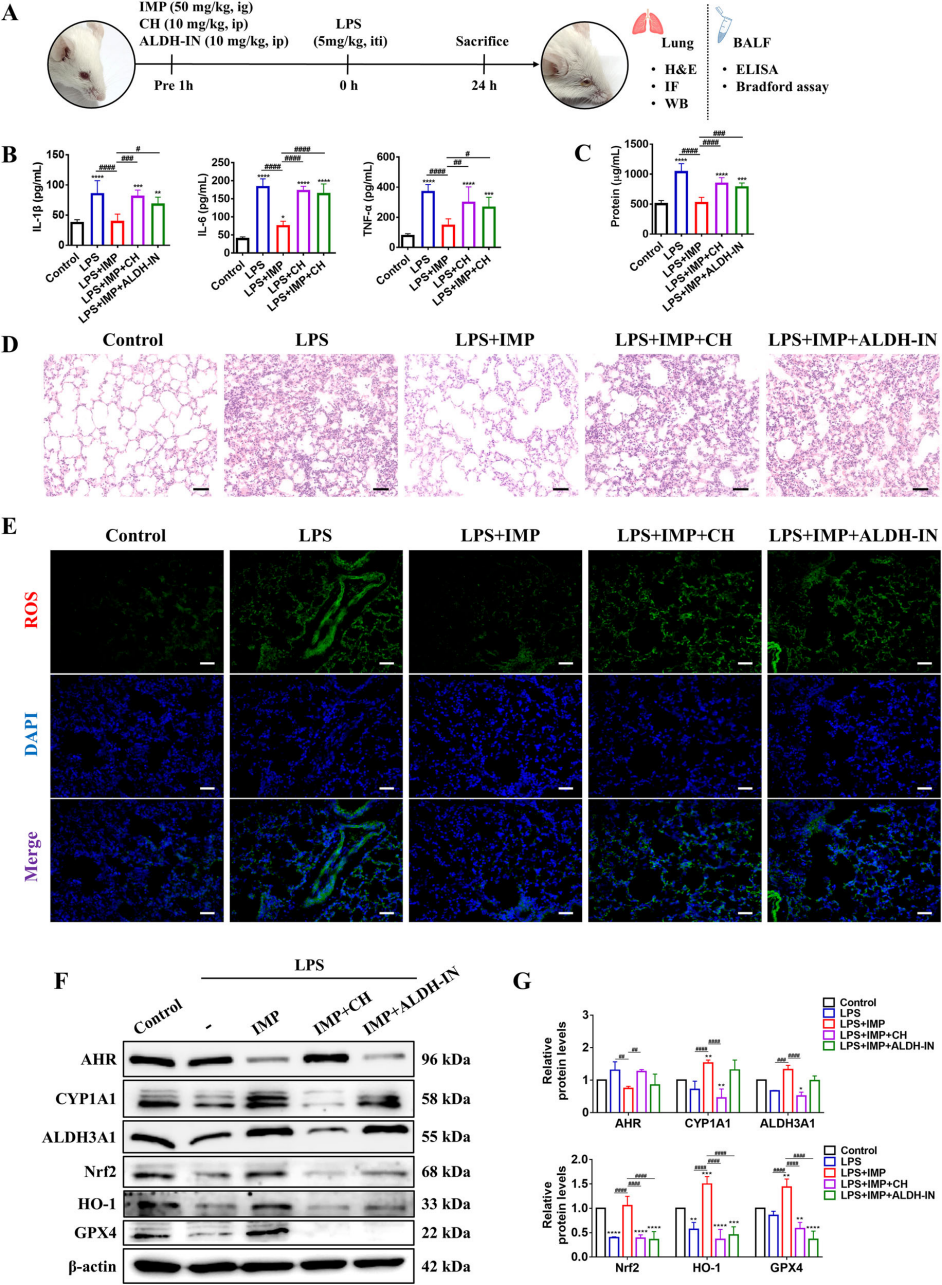
AHR or ALDH3A1 inhibitors abolish the protective effect of IMP in LPS-induced ALI mice. **A** Schematic diagram of IMP, CH, and ALDH-IN treatment of LPS-induced ALI mice. **B** BALF concentrations of IL-1β, IL-6, and TNF-α. **C** Total protein concentrations in BALF. **D** Representative H&E‑stained lung tissue sections (scale bar = 50 μm). **E** Representative ROS immunofluorescence staining of lung tissue (scale bar = 50 μm). *n* = 6 in all assays depicted in (**A**–**E**). **F**, **G** Western blotting analysis of AHR, CYP1A1, ALDH3A1, Nrf2, HO-1, and GPX4 proteins in lung tissue (*n* = 3). Abbreviations: ig intragastric gavage, ip intraperitoneal injection, iti intratracheal instillation. Data are expressed as mean ± SD. *p < 0.05, **p < 0.01, ***p < 0.001, ****p < 0.0001, versus control group; ^#^*p* < 0.05, ^##^*p* < 0.01, ^###^*p* < 0.001, ^####^*p* < 0.0001, versus LPS + IMP group.

In the final part of this study, to uncover whether IMP’s therapeutic effects on ALI in vivo were AHR/ALDH3A1-dependent, we pharmacologically inhibited endogenous AHR (using the antagonist CH) and ALDH3A1 (using the inhibitor ALDH-IN) in ALI mice (Fig. 8A). As hypothesized, co-treatment with IMP and either inhibitor substantially abrogated IMP’s ameliorative effects on LPS-induced pulmonary inflammation, histopathological damage, and oxidative stress in ALI mice, as evidenced by elevated inflammatory mediators (IL-1β, IL-6, and TNF-α) and total protein concentration in BALF (Fig. 8B, C), histopathological manifestations of alveolar collapse and thickened septal walls (Fig. 8D), and excessive ROS accumulation in lung tissue (Fig. 8E). Next, we examined the effects of CH and ALDH-IN on the AHR/ALDH3A1 and Nrf2/HO-1/GPX4 signaling pathways in ALI mouse lung tissue. Consistent with the in vitro studies (Figs. 3H, I and 4K, L), CH significantly inhibited IMP-induced AHR activation (downregulating its marker CYP1A1) and reversed the IMP-mediated upregulation of ALDH3A1, Nrf2, HO-1, and GPX4. In contrast, ALDH-IN blocked IMP-induced activation of the Nrf2/HO-1/GPX4 pathway without affecting AHR activation (Fig. 8F, G). This further proved that ALDH3A1 was critical for the regulation of the Nrf2/HO-1/GPX4 axis by IMP-activated AHR. Collectively, these findings conclusively establish the AHR/ALDH3A1 axis as a pivotal and non-redundant mechanism underpinning IMP’s anti-ALI efficacy.

## Discussion

Pulmonary immunity is a multifaceted process essential for responding to pathogenic threats. The initial lung injury triggers an inflammatory response, which, if not properly regulated, can lead to compensatory anti-inflammatory responses that result in tissue damage and disease progression [35]. AHR is expressed in multiple pulmonary cell types, including macrophages, type II alveolar cells, and endothelial cells. It serves as a sensor for pathogen-derived toxic factors in the lungs and is critical for host responses to environmental pollutants, bacterial infections, and viral challenges [36, 37]. Studies have demonstrated that AHR deficiency increased susceptibility to *P. aeruginosa* and *M. tuberculosis* in mice, whereas AHR activation modulated antibacterial defenses and cytokine production [38]. Another study revealed that AHR activation enhanced pulmonary adaptation to hyperoxia in rats, thereby ameliorating hyperoxic lung inflammation and injury [39]. Furthermore, in a cigarette smoke exposure model, AHR knockout mice exhibited significantly increased TNF-α and IL-6 concentrations in BALF, accompanied by exacerbated pulmonary inflammation and neutrophilic response [40]. These study findings suggest that AHR may be a potential therapeutic target for ALI, although further validation is required.

In addition, existing research indicates that IMP, as a natural coumarin compound, improves ALI by inhibiting inflammation, yet its biological mechanisms remain largely unexplored. To our knowledge, this study is the first to demonstrate that IMP triggers AHR activation in both lung tissues and lung epithelial cells. Extending these findings, our results further demonstrated that IMP-activated AHR alleviated LPS-induced inflammation, oxidative stress, and epithelial barrier damage in ALI models in vitro and in vivo. Specifically, AHR activation suppressed the secretion of inflammatory mediators IL-1β, IL-8, and TNF-α by inhibiting NF-*κ*B activation; it also lowered ROS levels by stimulating the Nrf2/HO-1 axis and maintained epithelial barrier stability by upregulating E-cadherin and Occludin. The observed reversal of these therapeutic benefits by AHR inhibitors substantiates the causal relationship. Collectively, these findings not only validate AHR as a promising therapeutic target for ALI management but also elucidate the pharmacological mechanism by which IMP ameliorates ALI through AHR activation.

The physiological impact of AHR activation by ligands is controversial due to their opposing effects. Beneficial ligands such as 6-formylindolo (3,2-b) carbazole, Glyteer, and coal tar enhance skin barrier function and suppress inflammation by activating AHR. CYP enzymes play a critical role in this process by enabling transient and moderate AHR activation [41, 42]. Conversely, dysregulated ligands such as particulate matter, polycyclic aromatic hydrocarbons, and 2,3,7,8-tetrachlorodibenzo-p-dioxin cause sustained AHR signaling, triggering severe inflammation and adverse health outcomes [43, 44]. Thus, the ligand metabolism mediated by CYP1A1 serves as an important negative feedback mechanism. Our study revealed that IMP induced AHR nuclear translocation and transcriptional activity, leading to moderate and transient activation rather than sustained activation. This was confirmed by IMP-triggered CYP1A1 or AHRR upregulation in LPS-stimulated A549 cells and mouse lung tissue, indicating AHR activation. Concurrent AHR protein downregulation suggested the initiation of the AHR negative feedback loop. Mechanistically, elevated CYP1A1 may degrade the AHR ligand IMP, while AHRR destabilizes AHR-ARNT complexes, collectively mitigating toxicity from sustained activation. Importantly, IMP demonstrated therapeutic potential against ALI in both in vivo and in vitro models through moderate AHR activation. While novel biologics may improve efficacy, oral bioavailability remains a critical clinical advantage. Thus, developing safe oral IMP formulations represents a viable therapeutic approach for ALI.

Ferroptosis, an iron-dependent form of cell death driven by lipid peroxidation, contributes to multiple organ injuries and degenerative pathologies [45]. Recent studies have demonstrated that activated AHR alleviates hepatic ferroptosis during ischemia/reperfusion injury by modulating the STAT3-HO-1/COX-2 axis [20]; however, the role of AHR in regulating ferroptosis in ALI remains largely unexplored. In this research, LPS was utilized to trigger ferroptosis in lung epithelial cells during ALI, and the AHR inhibitor CH was used to block IMP-mediated AHR activation. The results showed that CH not only negated IMP’s effects on Fe^2+^, ROS, and lipid peroxidation levels but also significantly prevented the IMP-induced upregulation of Nrf2, HO-1, and GPX4 proteins, supporting a protective mechanism in which AHR activation inhibited ferroptosis in lung epithelial cells via the Nrf2/HO-1/GPX4 pathway. Additionally, while previous studies have established an interaction between ALDH3A1 and antioxidant pathways [46, 47], its involvement in ferroptosis regulation remains unreported. Our data indicated that ALDH3A1 positively regulated the Nrf2/HO-1/GPX4 axis to suppress ferroptosis and functioned as a critical downstream target of AHR signaling. This was evidenced by the prevention of IMP-induced ALDH3A1 upregulation by CH, and by the blockage of AHR-mediated ferroptosis inhibition by ALDH-IN (a specific ALDH3A1 inhibitor) via the Nrf2/HO-1/GPX4 axis, along with the attenuation of lung protection in both in vitro and in vivo models. Collectively, IMP showed therapeutic potential against lung epithelial cell ferroptosis in ALI, with the AHR/ALDH3A1 axis representing a novel regulatory mechanism.

AHR has been widely recognized as a therapeutic target for various barrier organ diseases [11, 26]. In intestinal epithelial cells, AHR activation improves intestinal barrier function by maintaining tight junction proteins (ZO-1, Occludin, and Claudin-1) and alleviates inflammation by inhibiting NF-*κ*B, thereby improving inflammatory bowel disease [48]. Similarly, AHR activation in keratinocytes restores the skin barrier by upregulating epidermal differentiation genes (loricrin and involucrin) and inhibits oxidative stress via Nrf2 signaling, thereby alleviating skin disorders like psoriasis and atopic dermatitis [41, 42]. Emerging evidence suggests that IMP holds promise in mitigating colitis and psoriasis [49, 50]. However, current studies are limited, underscoring the necessity to elucidate the underlying mechanisms. In this study, we confirmed that IMP was a direct binder and agonist of AHR. Specifically, the luciferase reporter assay demonstrated its agonistic activity toward AHR (EC_50_ = 2.097 μM), molecular docking identified key binding residues, and MST produced a Kd value of 1.36 μM for their interaction. Furthermore, we demonstrated that IMP activated AHR in lung tissue in vivo. Given AHR’s broad distribution in barrier organs, our findings provide mechanistic insight into how IMP may modulate autoimmune diseases, like psoriasis and colitis, through tissue-specific AHR activation pathways.

Accumulating evidence suggests that Tapi functions as a novel regulator of AHR, capable of binding to and activating AHR in diverse cell types, and conferring anti-inflammatory, antioxidant, and barrier-protective effects [51]. In May 2022, 1% Tapi cream received approval in the United States as a topical therapy for plaque psoriasis in adults [16]. Notably, the topical application of Tapi is generally well tolerated with minimal systemic exposure. However, current evidence does not support its oral administration [52, 53]. Earlier research has emphasized the therapeutic promise of AHR ligands for treating various inflammatory lung conditions, including asthma, silicosis, and chronic obstructive pulmonary disease [54, 55]. Nevertheless, the precise role of Tapi as an AHR ligand in ALI treatment remains unclear, highlighting the need for innovative approaches to address this knowledge gap. In this study, Tapi was administered locally to the lungs of mice by tracheal nebulization. Tapi significantly enhanced AHR activity in the lungs of ALI mice, resulting in improved lung pathology, restored lung function, and reduced inflammatory damage. These findings underscore the potential of Tapi’s localized precision therapy in targeting AHR for ALI treatment. However, further preclinical and clinical investigations are warranted to assess the therapeutic efficacy of aerosolized Tapi delivery in ALI treatment regimens.

## Conclusion

**Fig. 9:**
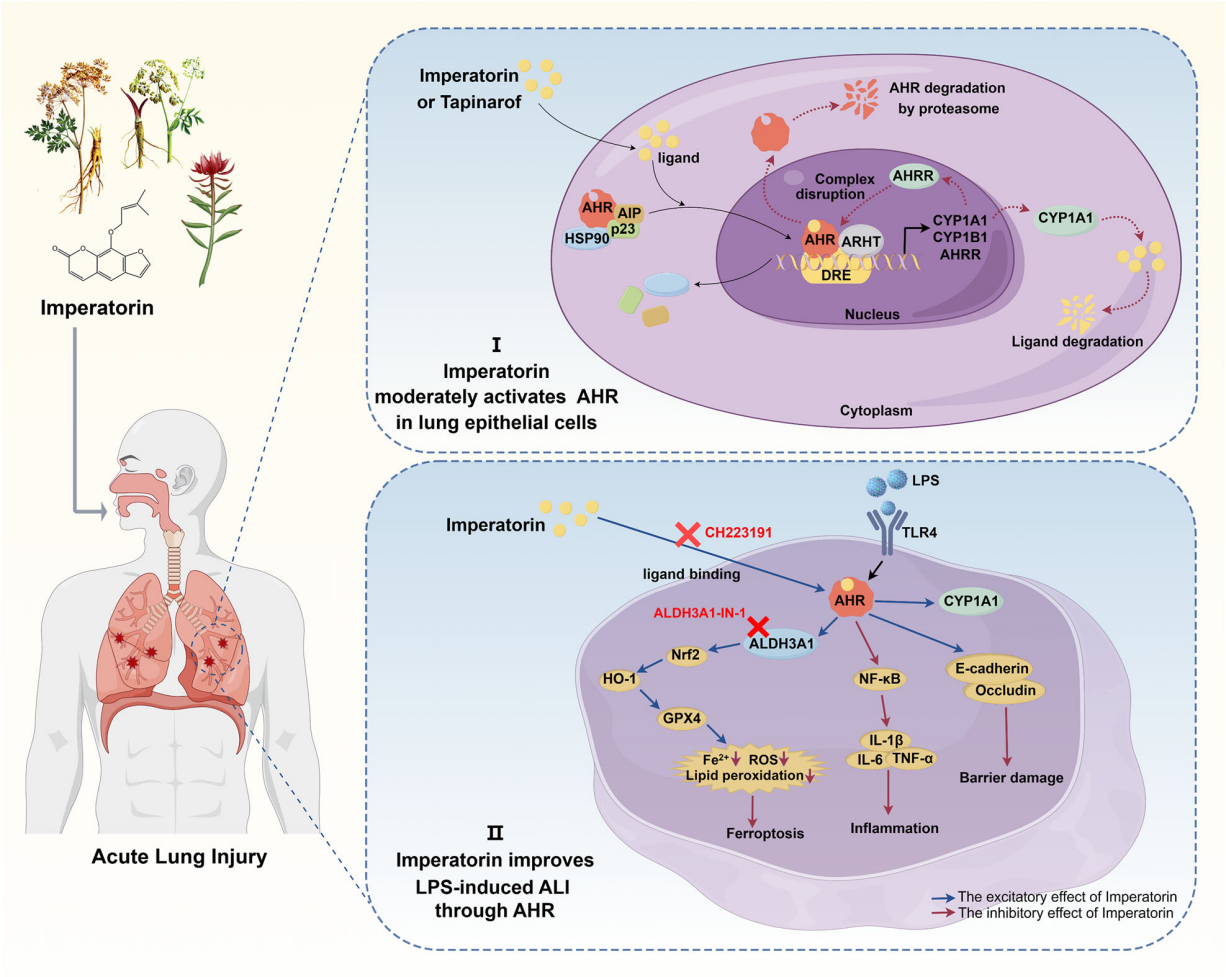
The proposed potential mechanism of IMP as an AHR agonist in the prevention and treatment of ALI.

In summary, our findings establish the AHR/ALDH3A1 axis as a novel therapeutic target for mitigating ferroptosis in ALI. IMP, functioning as a natural AHR agonist, promotes moderate AHR activation and nuclear translocation within lung epithelial cells. This leads to ALDH3A1 upregulation, subsequently activating the Nrf2/HO-1/GPX4 pathway to suppress LPS-induced ferroptosis. Furthermore, IMP-mediated AHR activation alleviates inflammation and preserves epithelial barrier integrity in ALI by modulating NF-*κ*B, E-cadherin, and Occludin. Together, these results establish IMP as a promising candidate for ALI therapy (Fig. 9).

## Materials and methods

### Reagents

Imperatorin (purity ≥ 98%) was provided by Herbpurify (Chengdu, China). Tapinarof, CH223191, and ALDH3A1-IN-1 were provided by MedChemExpress (NJ, USA). LPS was purchased from Sigma-Aldrich (MO, USA). RPMI 1640 and DMEM were bought from HyClone (UT, USA). Fetal bovine serum and penicillin/streptomycin were obtained from Gibco (NY, USA). Primary antibodies against AHR (#A22464), CYP1A1 (#A22182), LaminB1 (#A11495) and ABflo^TM^ 594-conjugated AffiniPure goat anti-rabbit IgG (H + L) (#AS074) were obtained from ABclonal (Wuhan, China). Primary antibodies against ALDH3A1 (#DF8502), HO-1 (#AF5393), GPX4 (#DF6701), E-cadherin (#AF0131), β-actin (#AF7018) and secondary antibodies were obtained from Affinity (OH, USA). Nrf2 (#YM4294) and Occludin (#YN2865) were obtained from ImmunoWay (TX, USA). NF-*κ*B (#A66433) and *p*-NF-*κ*B (#A52820) were purchased from Nature Biosciences (Zhenjiang, China). Horseradish peroxidase-conjugated secondary antibodies were obtained from Zsbio (Beijing, China).

### Cell culture and viability assay

A549 cells were sourced from the American Type Culture Collection (VA, USA), and HepG2-Lucia AHR reporter cells were sourced from InvivoGen (Toulouse, France). Cell maintenance was performed using RPMI 1640 (A549 cells) or DMEM (HepG2-Lucia AHR reporter cells) medium containing 10% fetal bovine serum and 1% penicillin/streptomycin at 37 °C with 5% CO₂. All cells were verified to be free of mycoplasma. Following the seeding of A549 cells (3 × 10³/well) in 96-well plates, the MTT assay (Biosharp, China) was performed to quantify viability after a 24-h treatment with IMP. The results were measured at 570 nm using a ReadMax 1500 instrument (Flash, China).

### RNA-seq

Following a 24 h exposure of A549 cells to DMSO or IMP, total RNA was extracted using TRIzol. After quality inspection, cDNA was synthesized with dUTP replacing dTTP, followed by terminal repair and dA-tailing. Adapter ligation and PCR amplification were used to construct sequencing libraries, which underwent 150-bp paired-end sequencing on an Illumina NovaSeq. Raw data quality was verified using FastQC, and differential expression analysis between the sample sets was performed using DESeq2. R software was used to create heatmaps and volcano plots of DEGs. Subsequently, functional enrichment of DEGs was performed using the KEGG, GO, Reactome, and DO databases.

### Immunofluorescence assay

Immunofluorescence was used to detect AHR nuclear translocation in A549 cells treated with IMP or Tapi for 24 h. The cells were fixed in 4% paraformaldehyde for 15 min, followed by permeabilization using 0.3% Triton X-100 (Beyotime, China) for 15 min, washed with phosphate-buffered saline (PBS) and blocked with 10% bovine serum albumin (Beyotime, China) for 1 h. The cells were then incubated with a primary anti-AHR antibody (1:100) and an ABflo™ 594-conjugated secondary antibody (1:200). Nuclear staining was performed using 10 μg/mL DAPI (Solarbio, China). A TCS SP8 confocal microscope (Leica, Germany) was used for imaging.

### Western blotting

A549 cells were treated with IMP, Tapi, LPS, the AHR inhibitor CH, or the ALDH3A1 inhibitor ALDH-IN for 24 h, with a control group included. Proteins were obtained from both the nucleus and cytoplasm utilizing the Nuclear and Cytoplasmic Protein Extraction Kit (Beyotime, China). Total protein was isolated using RIPA lysis buffer. Protein concentrations were determined using the Bradford assay (Beyotime, China). After quantification, the samples were mixed with SDS loading buffer and heated to 100 °C for 5 min. Proteins were separated on 10% SDS-PAGE gels and subsequently transferred to PVDF membranes (MerckMillipore, USA). The membranes were incubated with primary antibodies overnight at 4 °C, including AHR (1:1000), CYP1A1 (1:1000), ALDH3A1 (1:1000), Nrf2 (1:1000), HO-1 (1:1000), GPX4 (1:1000), NF-*κ*B (1:1000), *p*-NF-*κ*B (1:1000), E-cadherin (1:1000), Occludin (1:1000), and β-actin (1:1000). Subsequently, the membranes were incubated with secondary antibodies (1:5000) at 37 °C for 1 h. Protein bands were visualized using a chemiluminescence system (Abbkine, USA), and densitometric analysis was conducted using ImageJ software.

### qRT-PCR

Total RNA was isolated from cells using the Cell Total RNA Separation Kit (FOREGENE, China) and underwent Nanophotometer quantification (Implen, Germany), followed by cDNA synthesis with the RT Easy™ II kit (FOREGENE, China). qRT-PCR analysis was performed using THUNDERBIRD SYBR Green chemistry (Toyobo, Japan) on an Archimed X4 system (ROCGENE, China). Relative gene expression was calculated using the ΔΔCT method with GAPDH normalization. The specific primers used are shown in Table 1.

**Table 1:** Primer sequences of the target genes.

Target genes	Forward primer (5–3′)	Reverse primer (3–5′)
Human *AHR*	ATCACCTACGCCAGTCGCAAG	AGGCTAGCCAAACGGTCCAAC
Human *CYP1A1*	TCGGCCACGGAGTTTCTTC	GGTCAGCATGTGCCCAATCA
Human *AHRR*	GCGCCTCAGTGTCAGTTACC	GAAGCCCAGATAGTCCACGAT
Human *ILIB*	GCCAGTGAAATGATGGCTTATT	AGGAGCACTTCATCTGTTTAGG
Human *CXCL8*	GACCACACTGCGCCAACAC	CTTCTCCACAACCCTCTGCAC
Human *Tnf*	CCTCTCTCTAATCAGCCCTCTG	GAGGACCTGGGAGTAGATGAG
Human *GAPDH*	GAGAAGGCTGGGGCTCATTT	AGTGATGGCATGGACTGTGG

### Luciferase reporter assay

HepG2-Lucia™ AHR reporter cells were seeded at 1 × 10^4^ cells/well in 96-well plates and treated for 24 h with IMP, Tapi, or CH, whereas control group received the DMSO vehicle. Luminescence detection was performed using QUANTI-Luc™ (InvivoGen, rep-qlc2) and the LumiStation 1800 instrument (Flash, China). Luminescence signals were normalized to the control group. EC_50_ values were calculated using least-squares curve fitting.

### Molecular docking

The MOL2 format file of IMP molecule was sourced from the TCMSP database, and the 3D structure of the AHR protein was obtained from the Protein Data Bank (ID: 7ZUB). Protein receptors were prepared by removing ligands and water molecules, followed by molecular docking with IMP using Discovery Studio.

### MST assay

Recombinant human AHR protein was obtained from MedChemExpress (NJ, USA). The protein was fluorescently labeled with the His-Tag Labeling Kit RED-tris-NTA 2nd Generation (NanoTemper, Germany) according to the manufacturer’s instructions. After labeling, the samples were loaded into MST capillaries (NanoTemper, Germany). The interaction between AHR and IMP or Tapi was measured using a Monolith NT.115 instrument (NanoTemper, Germany). Finally, the data were processed using MO-Affinity analysis software to calculate the binding constant (Kd).

### ROS assay

Intracellular superoxide anion and ROS levels were detected using ROS Assay Kit and DHE fluorescent probe (Beyotime, China), following the manufacturer’s standardized protocol.

### Fe^2+^ assay

Intracellular Fe^2+^ levels were measured utilizing the FerroOrange fluorescent probe (DOJINDO, Japan), in accordance with the guidelines provided by the manufacturer.

### Lipid peroxidation assay

Intracellular lipid peroxidation was measured using a Lipid Peroxidation Assay Kit with BODIPY 581/591 C11 (Beyotime, China), according to the manufacturer’s instructions.

### Wound healing assay

A549 cells were evenly distributed in a 6-well plate and cultured to 90% confluence. After making a standardized scratch using a 200-µL pipette tip, the detached cells were removed by washing with PBS three times. The cells were treated with LPS, IMP, or Tapi in serum-free RPMI 1640 medium. Microscopy was used to capture wound images at 0 h and 24 h post-scratching. The migration percentage was calculated as: [(A₀ − A₂₄)/A₀] × 100%, where A₀ and A₂₄ represent initial and 24 h scratch areas respectively.

### Animal experiments

Male BALB/c mice (18–20 g body weight) were sourced from Beijing HFK Bioscience Co., Ltd. (Beijing, China) and maintained in a specific pathogen-free environment and provided sterilized feed, bedding, and water. All animal experimental protocols were strictly conducted in accordance with the Guide for the Care and Use of Laboratory Animals. The research protocol was approved by the Animal Ethics Committee of Chengdu University of Traditional Chinese Medicine (ethics code: 2024-060).

After a 1-week acclimatization, the mice were randomly assigned to four groups: Control (4% DMSO + 16% PEG400 + 80% H_2_O) and IMP (25, 50, or 100 mg/kg) groups, with three mice per group. Lung tissues were harvested 1 h following oral administrations for Western blotting to determine the optimal IMP dose for activating AHR in subsequent pharmacodynamic experiments. After determining the IMP dose, the mice were randomly divided into six groups: Control, LPS (5 mg/kg) model, LPS (5 mg/kg) + IMP (50 mg/kg), LPS (5 mg/kg) + Tapi (5 μM), LPS (5 mg/kg) + IMP (50 mg/kg) + CH (10 mg/kg), and LPS (5 mg/kg) + IMP (50 mg/kg) + ALDH-IN (10 mg/kg), with six mice per group. IMP was administered intragastric gavage, CH and ALDH-IN were administered by intraperitoneal injection, and Tapi and LPS were delivered through intratracheal instillation using a pulmonary atomizing drug delivery device (TOW-INT, China). LPS was administered to all groups (excluding controls) 1 h post-treatment with IMP, Tapi, CH, or ALDH-IN. The control group received an equivalent volume of PBS to account for potential effects of the intratracheal instillation procedure itself. Any mice that died due to improper operation were excluded and supplemented the same amount to maintain group sizes. All mice were euthanized 24 h after LPS stimulation. Researchers were not blinded to the animal grouping.

### In vivo micro-CT imaging

The mice were anesthetized using 1% pentobarbital sodium, and pulmonary micro-CT imaging was performed using a Quantum GX system (PerkinElmer, USA). The scanning parameters were as follows: FOV, 36 mm; X-ray kV, 70 kV; X-ray μA, 80 μA; pixel size, 72 µm; and scanning time, 4 min. Pulmonary micro‑CT images were analyzed using Analyze 12.0 software, and 3D reconstructions were generated.

### Pulmonary function measurements

Pulmonary function was assessed using WBP (TOW-INT, China) before euthanizing the mice. Briefly, conscious mice were placed in a closed transparent chamber for 5 min to establish a stable respiratory baseline. The respiratory parameters of the unconstrained mice were then recorded for 2 min, including EF_50_, PEF, f, VT, MV, TI/TE, PenH, and Rpef. To minimize respiratory abnormalities due to anxiety, the assessments were conducted in a quiet environment with no visible other animals or personnel.

### Histological analysis

Lung tissues were harvested after 24 h of LPS stimulation. One portion was fixed in 4% paraformaldehyde, paraffin-embedded, sectioned, and stained with H&E. The sections were then examined by histopathological analysis using a NanoZoomer S60 system (Hamamatsu Photonics, Japan). The contralateral portion was embedded in OCT compound for cryosectioning and stained with the DCFH-DA fluorescent probe (Servicebio, China). ROS visualization was subsequently performed using a confocal microscope (Leica, Germany).

### Weight and lung W/D ratio

Following a 24 h LPS exposure, the lungs were harvested and weighed immediately to obtain the wet weight. Then, they were dried at 60 °C for 48 h before re-weighing to determine the dry weight. The lung W/D ratio was derived by dividing the wet weight by the dry weight.

### BALF assay

The mice were anesthetized using 1% pentobarbital sodium, blood samples were collected, and the lungs were lavaged with pre-cooled sterile PBS, which resulted in a recovery rate of over 90% of the BALF. Both the blood and BALF samples were centrifuged at 4 °C (12,000 rpm, 10 min) to obtain serum and BALF supernatant. Pro-inflammatory cytokine levels (IL-1β, IL-6, TNF-α) in both serum and BALF supernatant were measured using mouse-specific ELISA kits (Elabscience, China). Additionally, BALF supernatant protein concentrations were determined using the Bradford assay.

### Statistical analysis

All data are presented as mean ± standard deviation. Statistical analyses were conducted using SPSS 20.0 software, and data comparisons among groups were evaluated using one-way analysis of variance. A *p*-value of < 0.05 was regarded as statistically significant. Image analysis was conducted using ImageJ software, and graphs were created using GraphPad Prism 7 and FigDraw.

## Supplementary information

Original Western Blots

## Data Availability

Data will be made available on request.
