# Supplementary material for: ALDH3A1-dependent Nrf2/HO-1/GPX4 pathway supports AHR as a promising therapeutic target for ferroptosis and promotes imperatorin-mediated lung protection
Source: Cell Death Discov. 2026 Jan 9;12:16. doi: 10.1038/s41420-025-02860-8 (PMC12789433; doi:10.1038/s41420-025-02860-8)

Fig. 2C. Western blot bands of AHR and CYP1A1 in the cytoplasm of A549 cells exposed to Tapi or IMP for 24 h.

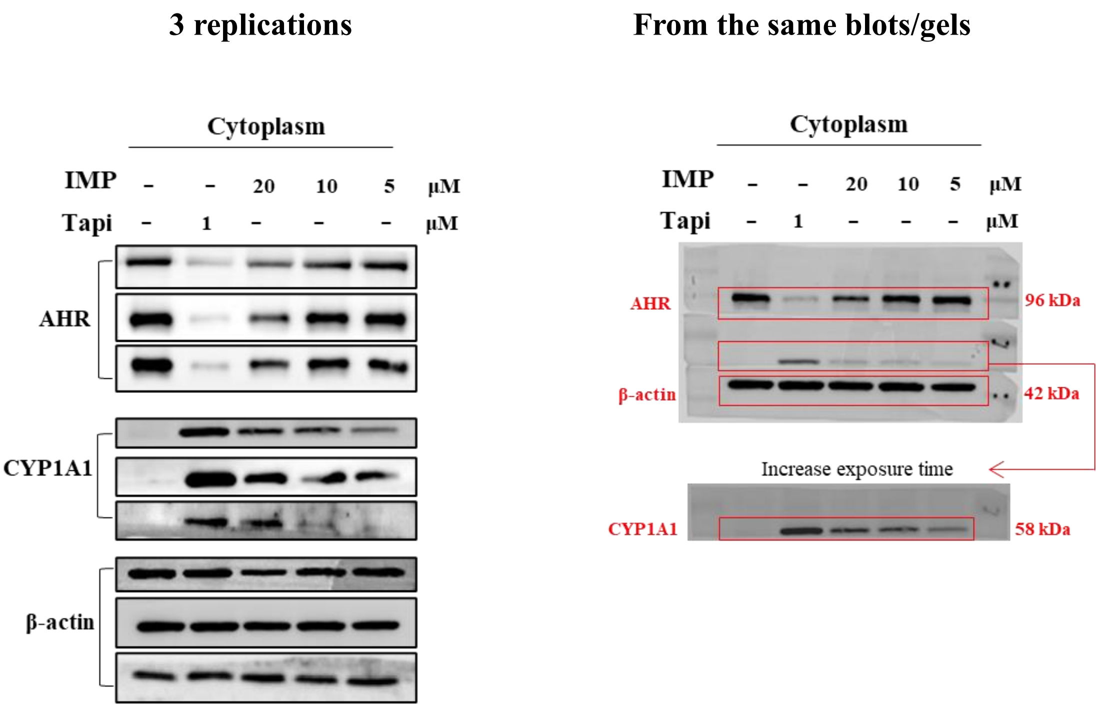

Fig. 2D. Western blot bands of AHR and CYP1A1 in the nucleus of A549 cells exposed to Tapi or IMP for 24 h.

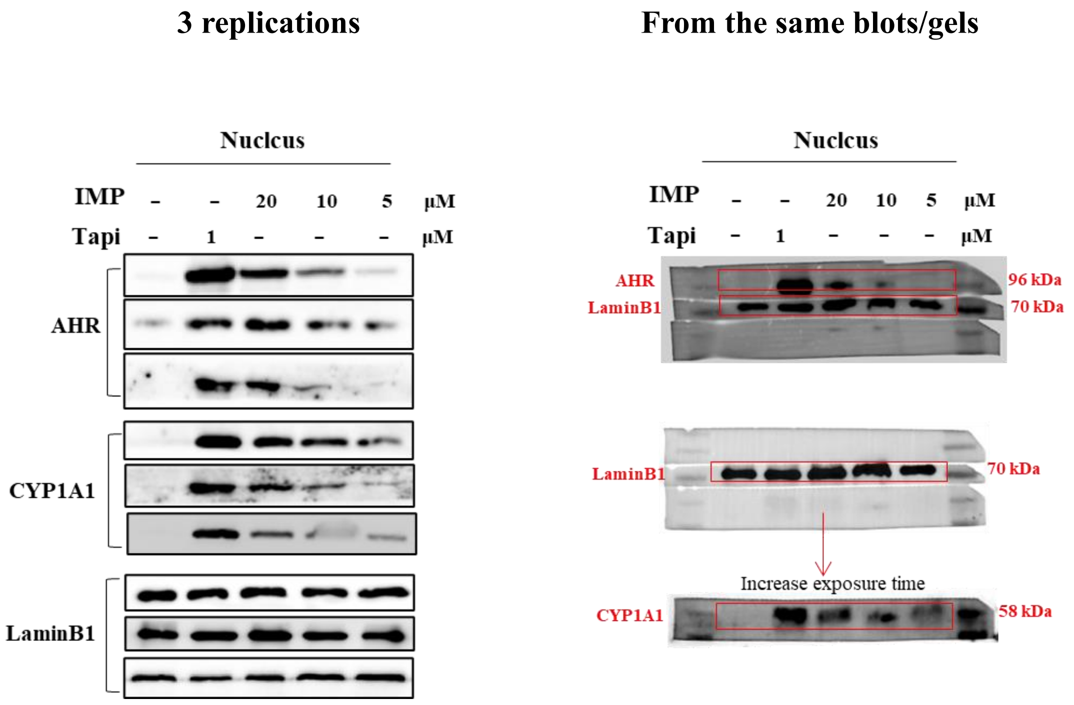

Fig. 2F. Western blot bands of AHR and CYP1A1 in A549 cells exposed to Tapi, CH or IMP for 24 h.

3 replications

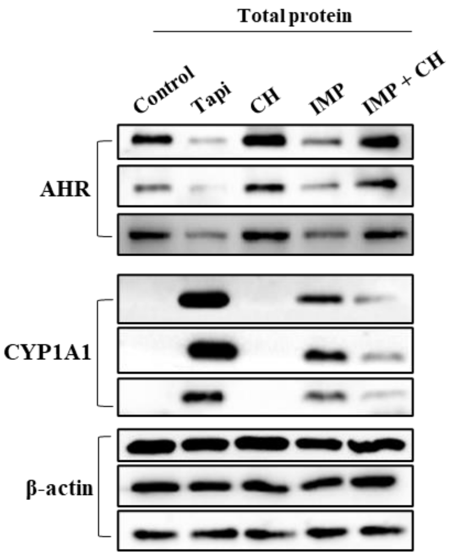

From the same blots/gels

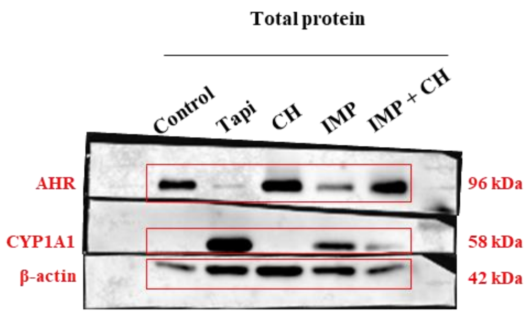

Fig. 3F. Western blot bands of AHR and CYP1A1 in LPS-induced A549 cells treated with IMP for 24 h.

3 replications

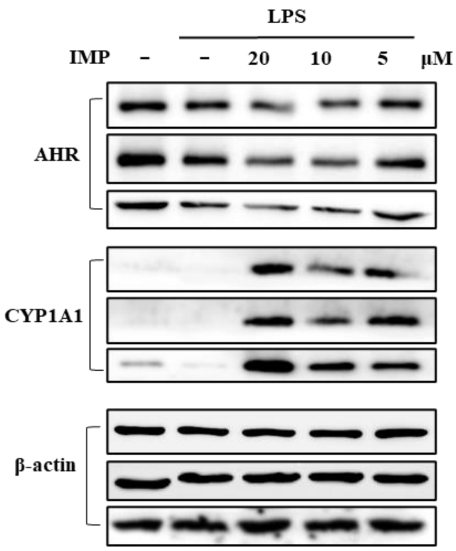

From the same blots/gels

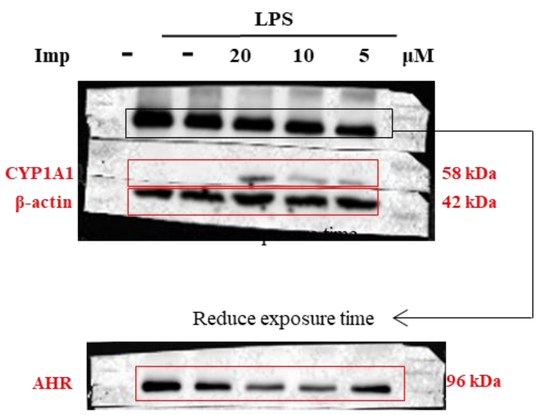

Fig. 3H. Western blot bands of AHR, CYP1A1, Nrf2, HO-1 and GPX4 in LPS-induced A549 cells treated with IMP for 24 h.

3 replications

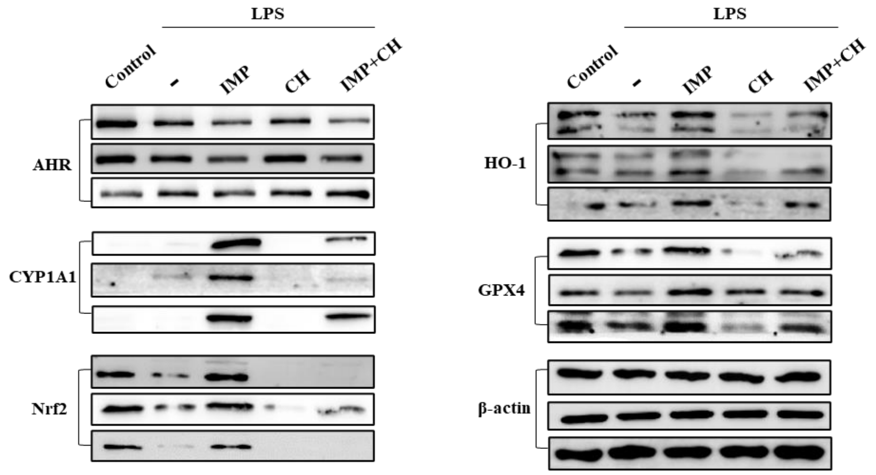

From the same blots/gels

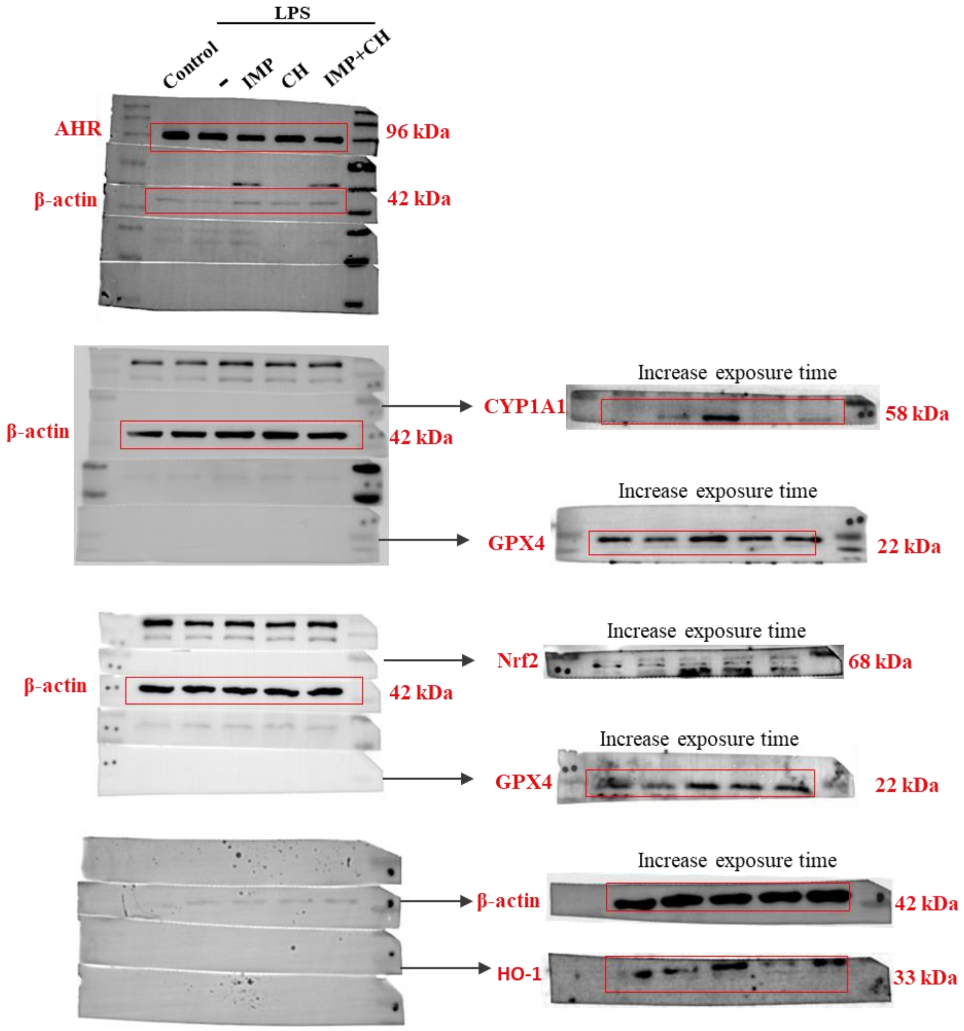

Fig. 4G. Western blot bands of AHR, CYP1A1, and ALDH3A1 after A549 cells were treated with Tapi or IMP for 24 h.

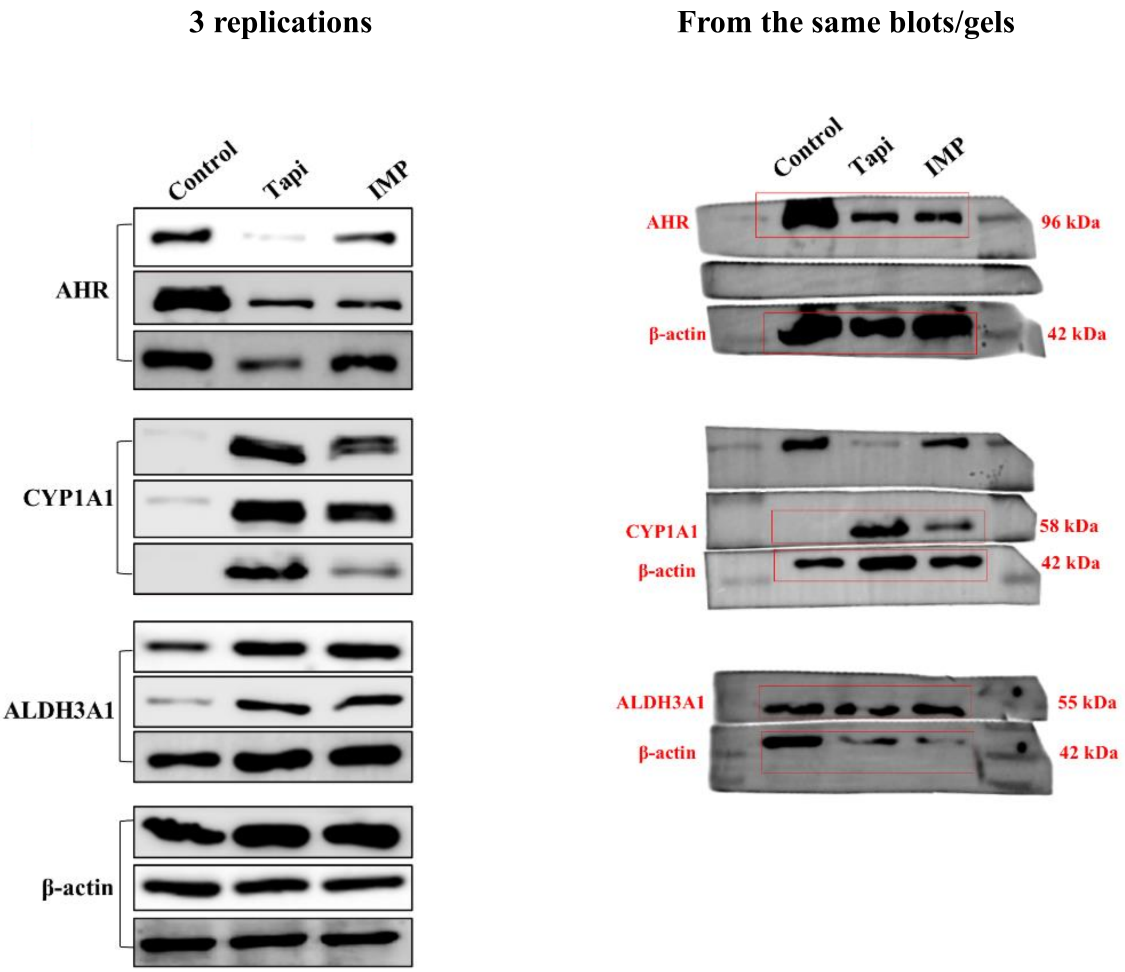

Fig. 4I. Western blot bands of AHR, CYP1A1, and ALDH3A1 after LPS-induced A549 cells were treated with Tapi or CH for 24 h.

3 replications

From the same blots/gels

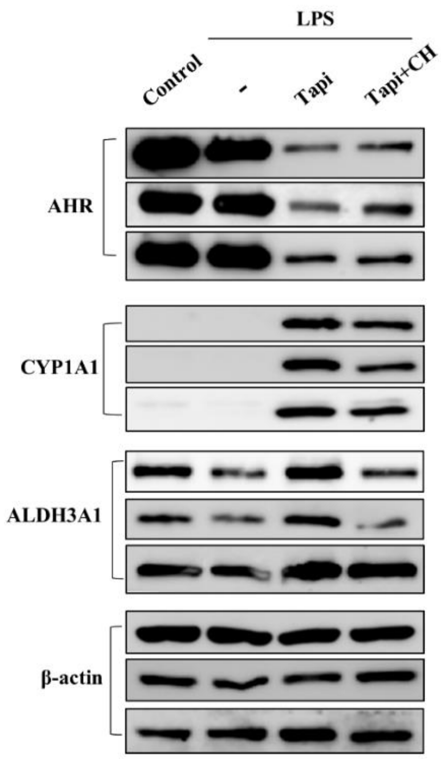

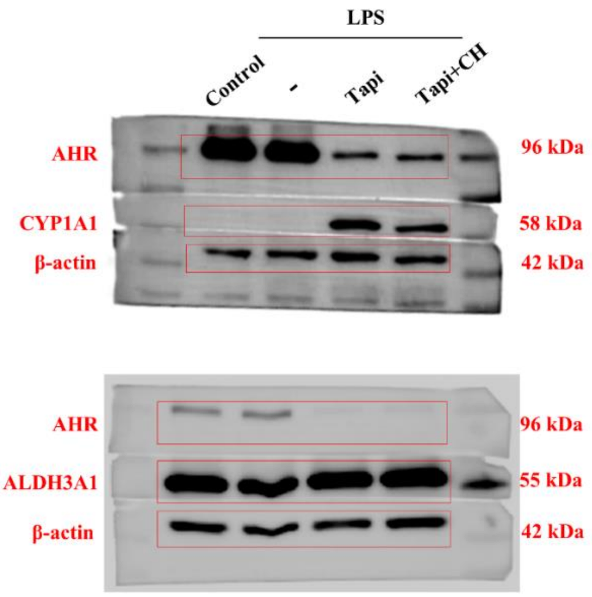

Fig. 4K. Western blot bands of AHR, CYP1A1, ALDH3A1, Nrf2, HO-1 and GPX4 after LPS-induced A549 cells were treated with IMP, CH or ALDH-IN for 24 h.

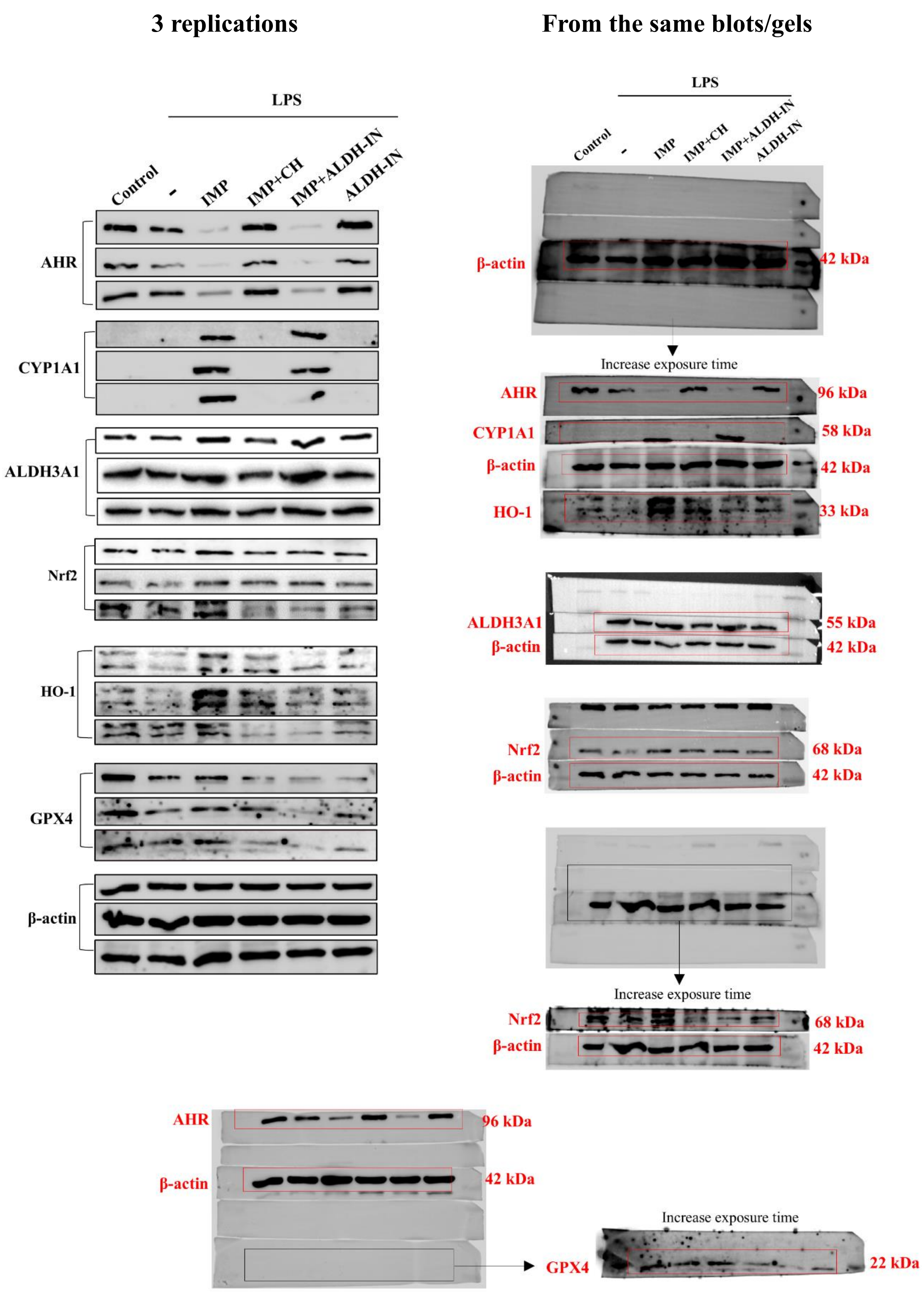

Fig. 5B. Western blot bands of NF-*κ*B and *p*-NF-*κ*B in LPS-induced A549 cells treated with IMP and CH for 24 h.

3 replications

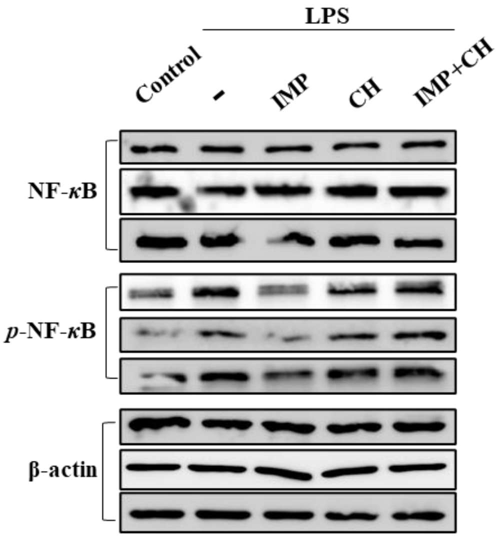

From the same blots/gels

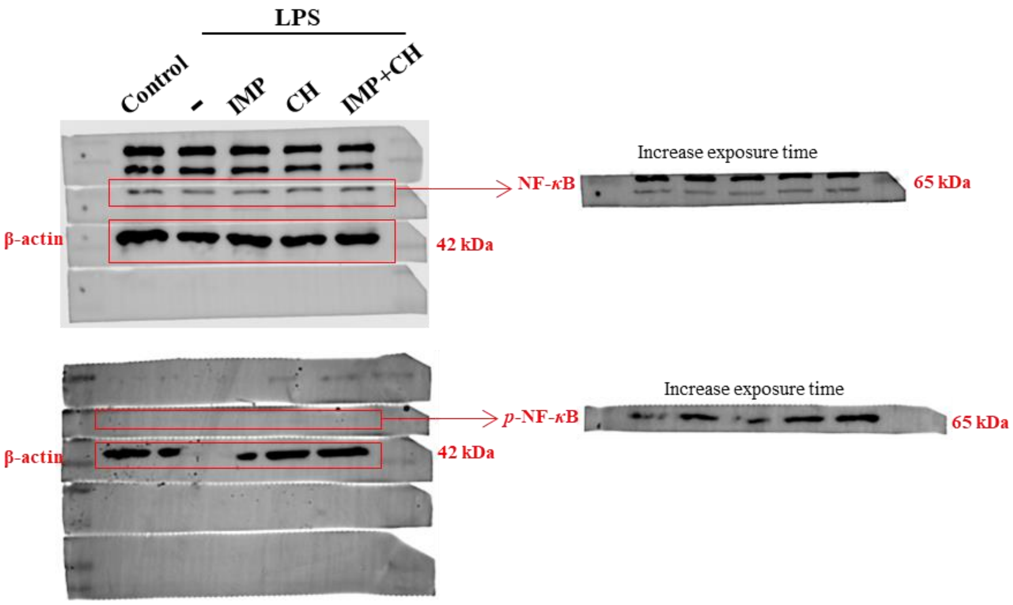

Fig. 5F. Western blot bands of E-cadherin and Occludin in LPS-induced A549 cells treated with IMP and CH for 24 h.

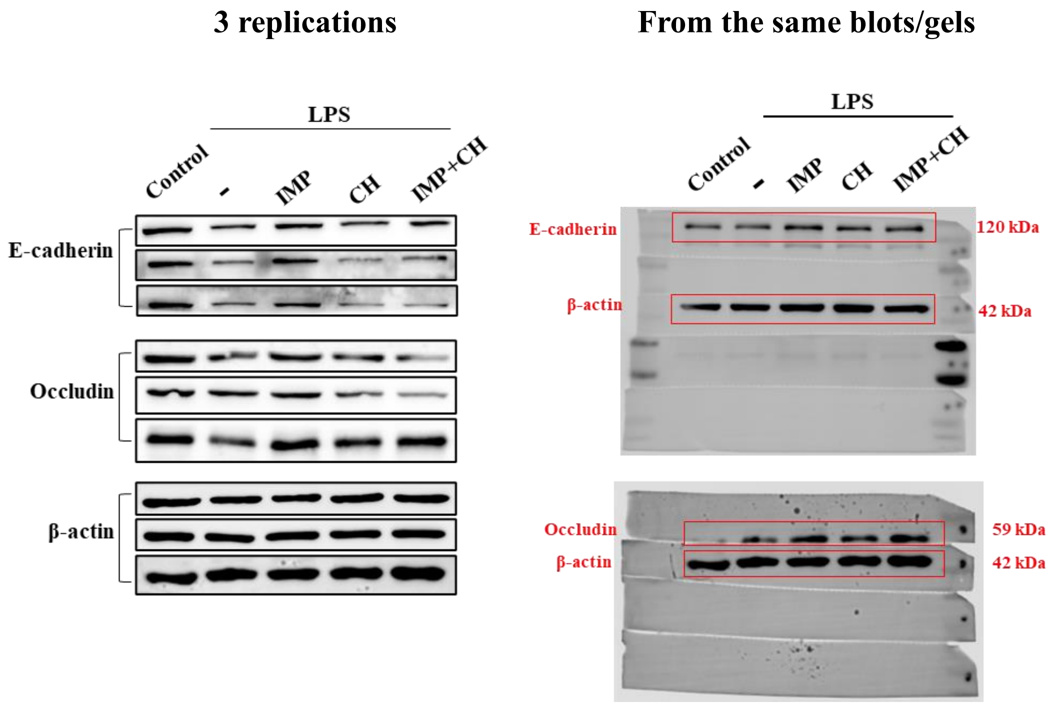

Fig. 6A. Western blot bands of CYP1A1 in the lung tissues of mice after oral administration of IMP for 1 h.

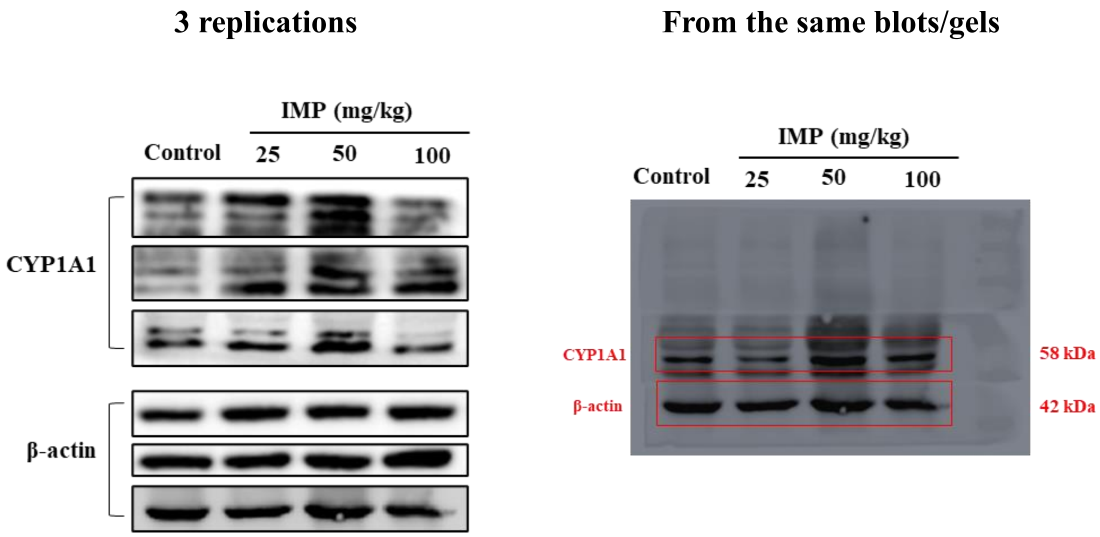

Fig. 7H. Western blot bands of AHR, CYP1A1, Nrf2, HO-1, and GPX4 in the lung tissues.

3 replications

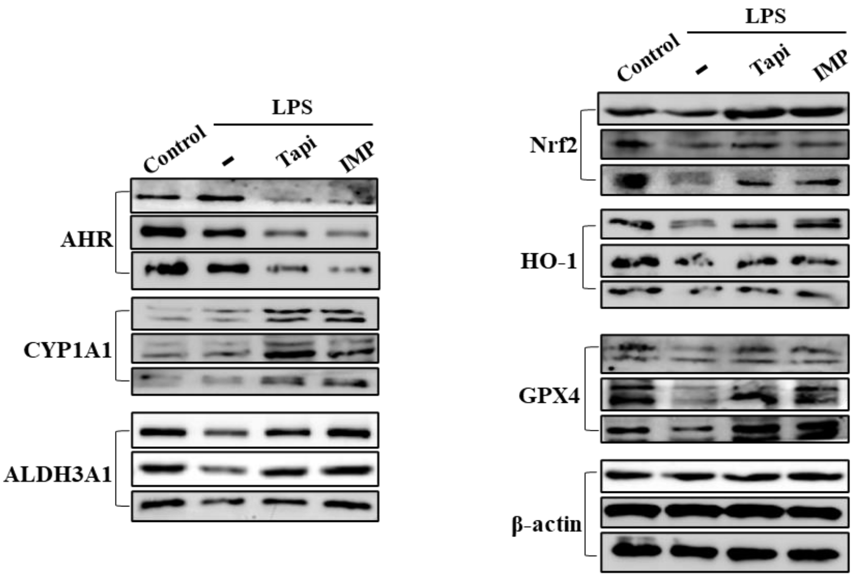

From the same blots/gels

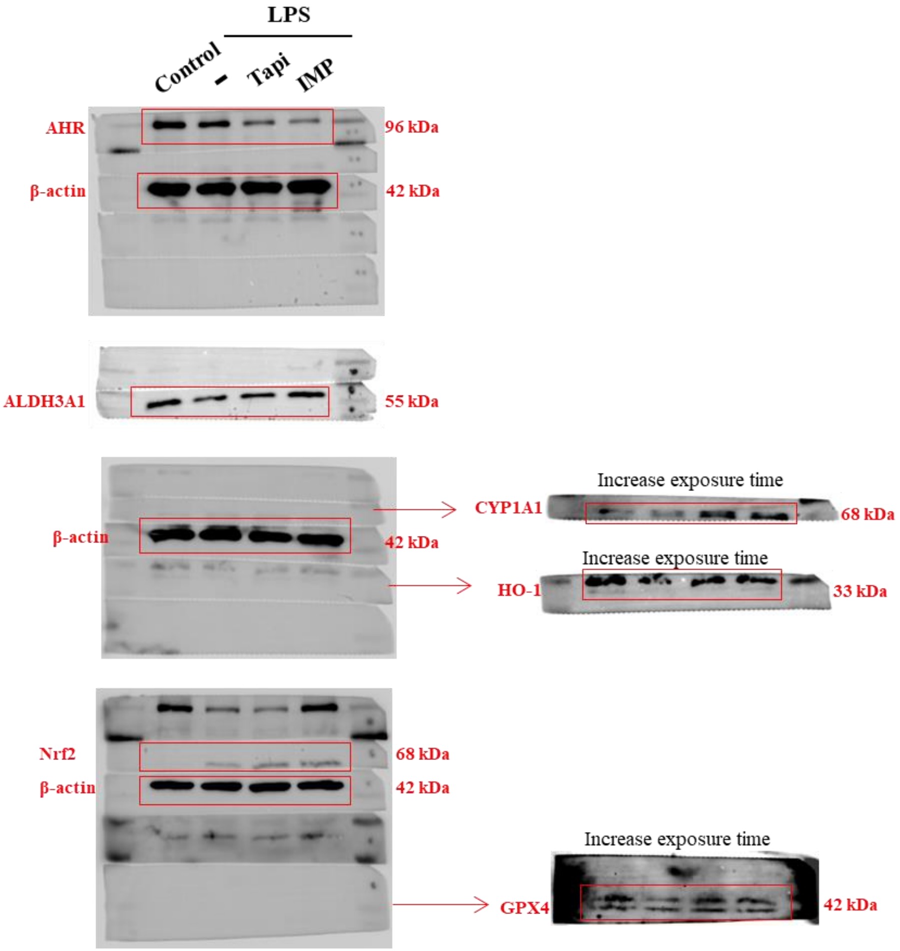

Fig. 8F. Western blot bands of AHR, CYP1A1, ALDH3A1, Nrf2, HO-1, and GPX4 in the lung tissues.

3 replications

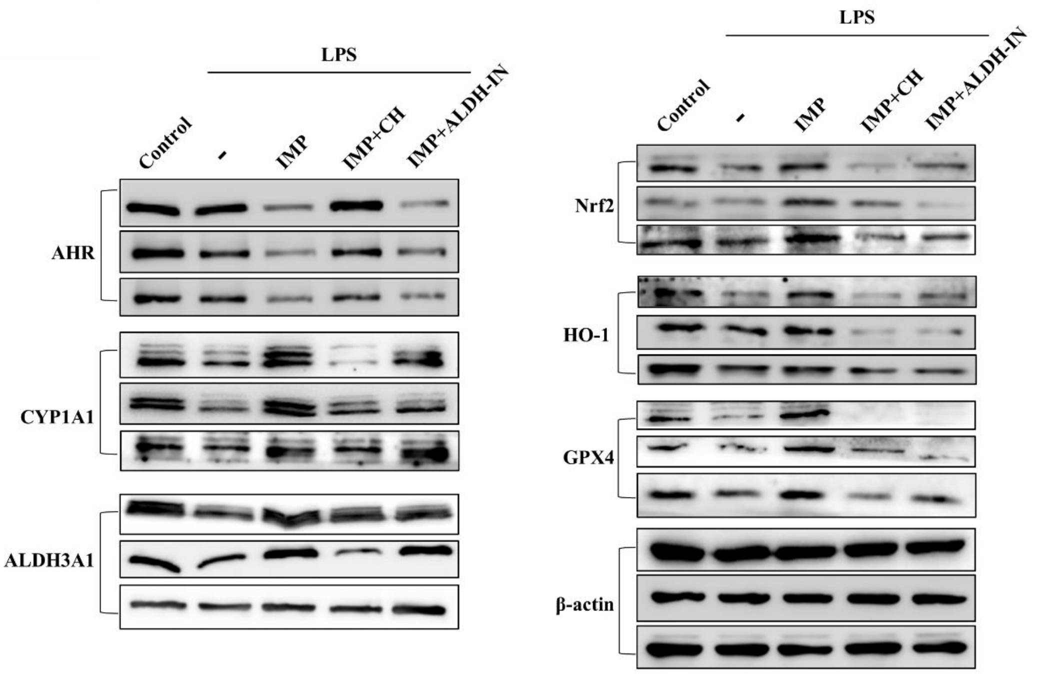

From the same blots/gels

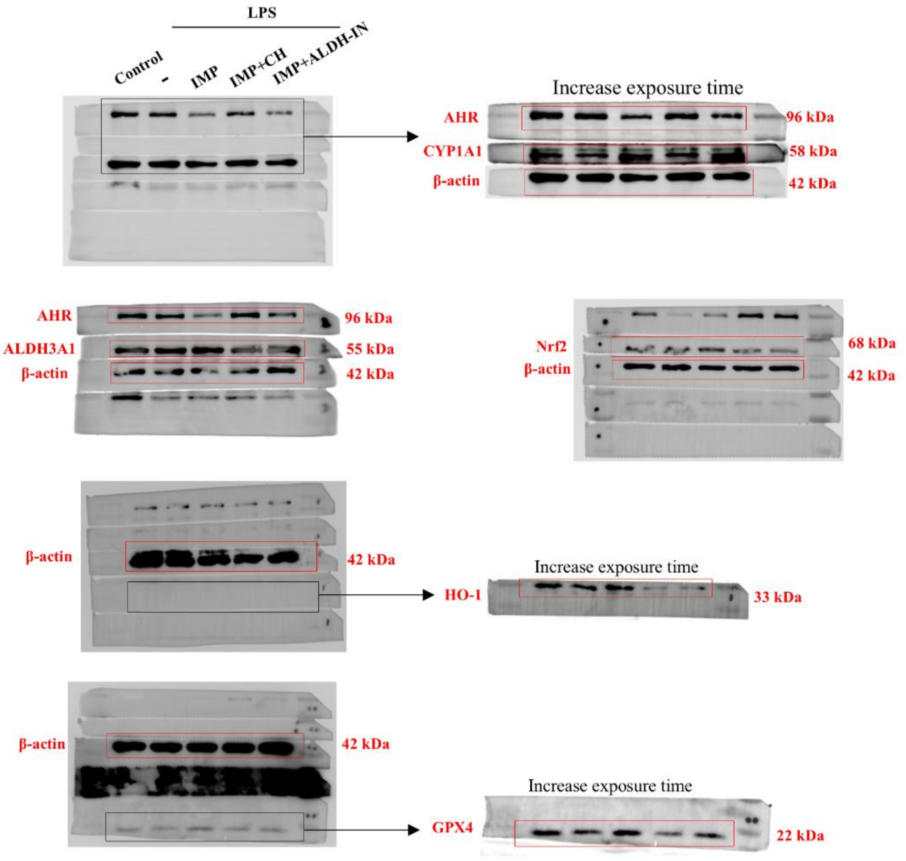

Supplement: Supplementary file 1 — Original Western Blots [file 41420_2025_2860_MOESM1_ESM.pdf]
